# Pre-Clinical Autoimmunity in Lupus Relatives: Self-Reported Questionnaires and Immune Dysregulation Distinguish Relatives Who Develop Incomplete or Classified Lupus From Clinically Unaffected Relatives and Unaffected, Unrelated Individuals

**DOI:** 10.3389/fimmu.2022.866181

**Published:** 2022-06-03

**Authors:** Melissa E. Munroe, Kendra A. Young, Joel M. Guthridge, Diane L. Kamen, Gary S. Gilkeson, Michael H. Weisman, Mariko L. Ishimori, Daniel J. Wallace, David R. Karp, John B. Harley, Jill M. Norris, Judith A. James

**Affiliations:** ^1^ Arthritis and Clinical Immunology Program, Oklahoma Medical Research Foundation, Oklahoma City, OK, United States; ^2^ Department of Epidemiology, Colorado School of Public Health, Aurora, CO, United States; ^3^ Department of Medicine, Oklahoma University Health Sciences Center, Oklahoma City, OK, United States; ^4^ Division of Rheumatology, Medical University of South Carolina, Charleston, SC, United States; ^5^ Division of Rheumatology, Cedars-Sinai Medical Center, Los Angeles, CA, United States; ^6^ Division of Rheumatic Diseases, University of Texas Southwestern Medical Center, Dallas, TX, United States; ^7^ US Department of Veterans Affairs Medical Center, Cincinnati, OH, United States; ^8^ Department of Pathology, Oklahoma University Health Sciences Center, Oklahoma City, OK, United States

**Keywords:** autoimmunity, systemic lupus erythematosus, autoantibodies, cytokines, pre-clinical disease, family studies, follow-up studies, risk assessment

## Abstract

Systemic lupus erythematosus (SLE) is propelled by pathogenic autoantibody (AutoAb) and immune pathway dysregulation. Identifying populations at risk of reaching classified SLE is essential to curtail inflammatory damage. Lupus blood relatives (Rel) have an increased risk of developing SLE. We tested factors to identify Rel at risk of developing incomplete lupus (ILE) or classified SLE vs. clinically unaffected Rel and healthy controls (HC), drawing from two unique, well characterized lupus cohorts, the lupus autoimmunity in relatives (LAUREL) follow-up cohort, consisting of Rel meeting <4 ACR criteria at baseline, and the Lupus Family Registry and Repository (LFRR), made up of SLE patients, lupus Rel, and HC. Medical record review determined ACR SLE classification criteria; study participants completed the SLE portion of the connective tissue disease questionnaire (SLE-CSQ), type 2 symptom questions, and provided samples for assessment of serum SLE-associated AutoAb specificities and 52 plasma immune mediators. Elevated SLE-CSQ scores were associated with type 2 symptoms, ACR scores, and serology in both cohorts. Fatigue at BL was associated with transition to classified SLE in the LAUREL cohort (*p≤0.01*). Increased levels of BLyS and decreased levels of IL-10 were associated with type 2 symptoms (p*<0.05*). SLE-CSQ scores, ACR scores, and accumulated AutoAb specificities correlated with levels of multiple inflammatory immune mediators (*p<0.05*), including BLyS, IL-2Rα, stem cell factor (SCF), soluble TNF receptors, and Th-1 type mediators and chemokines. Transition to SLE was associated with increased levels of SCF (*p<0.05*). ILE Rel also had increased levels of TNF-α and IFN-γ, offset by increased levels of regulatory IL-10 and TGF-β (*p<0.05*). Clinically unaffected Rel (vs. HC) had higher SLE-CSQ scores (*p<0.001*), increased serology (*p<0.05*), and increased inflammatory mediator levels, offset by increased IL-10 and TGF-β (*p<0.01*). These findings suggest that Rel at highest risk of transitioning to classified SLE have increased inflammation coupled with decreased regulatory mediators. In contrast, clinically unaffected Rel and Rel with ILE demonstrate increased inflammation offset with increased immune regulation, intimating a window of opportunity for early intervention and enrollment in prevention trials.

## 1 Introduction

Systemic lupus erythematosus (SLE) is a multifaceted autoimmune disease associated with chronic, underlying immune dysregulation. Altered immune pathways and the development of SLE-associated autoantibodies have been noted prior to the development of clinical disease, with continued expansion and accumulation as patients move toward disease classification (1, 2). Observed benefits of early intervention for patients at high risk of other autoimmune diseases such as type 1 diabetes mellitus (3) and rheumatoid arthritis (4) suggest that early intervention could also be particularly beneficial in SLE, where irreversible organ damage is often present by the time patients are diagnosed (5–8). Fundamental to successful early intervention is the identification of preclinical factors that signal and differentiate disease transition from states of latent autoimmunity that may never progress. This may be particularly true for relatives of SLE patients, who have an increased risk of developing SLE compared to the general population (9, 10).

Autoantibody specificities alone are insufficient to identify relatives at highest risk of developing lupus (11), as other forms of immune dysregulation both preface and coincide with autoantibody production to give rise to clinical sequelae and SLE transition (1, 2). Type I IFN (IFN-α) genetic polymorphisms and activity are associated with SLE pathogenesis (12) in lupus relatives (13), with enhanced IFN activity particularly associated with DNA- and RNA-protein binding autoantibody specificities (14, 15). In addition to type I IFN, multiple genes that contribute to activation of type II IFN (IFN-γ) pathways are associated with SLE (16, 17), with IFN-γ being among the earliest dysregulated mediators noted in pre-clinical SLE (1, 2), promoting a chronic pro-inflammatory cascade contributing to SLE disease pathogenesis (18, 19). Furthermore, IFN-γ can drive both type I IFN (20) and B-lymphocyte stimulator (BLyS) production (21–27). Bridging innate and adaptive immunity, IFN-γ perpetuates Th1-type adaptive cellular responses, recruiting cells to sites of inflammation by stimulating the secretion of such chemokines as MCP-1 (CCL2), MCP-3 (CCL7), MIG (CXCL9), and IP-10 (CXCL10) (20, 28–30). Another consistently detected pro-inflammatory mediator detected as patients transition to SLE (1, 11) and a marker of impending lupus disease flare (18, 19) is stem cell factor (SCF), associated with hematopoiesis, T-cell differentiation, and chemokine release (31, 32). Other immunoregulatory mechanisms, including levels of circulating IL-10 and TGF-β, also appear to be altered in SLE disease pathogenesis (1, 11, 18, 19).

Although immune dysregulation is a key precipitating factor to clinical disease development, affected individuals may or may not be aware of the ongoing immunological imbalance. Despite their sometimes difficult discernment, patient-reported symptoms are being increasingly recognized as a valuable focus to bridge the patient-provider disconnect noted in SLE (33, 34). A number of “type 2” manifestations noted in SLE that are unclear in origin and have an uncertain connection to underlying inflammation (33, 35), particularly fatigue, but also anxiety, depression, cognitive dysfunction/headaches, and sleep disturbances, are reported by patients early in disease development (36, 37). In addition, the connective tissue disease screening questionnaire (CSQ) was developed as a patient-reported screening tool for various connective tissue diseases (CTD), including SLE (38). Although validated in the general population (39, 40), the SLE portion of the questionnaire (SLE-CSQ) is based on ACR classification criteria for SLE and has the potential for identification of lupus relatives who may remain clinically unaffected vs. being at increased risk of developing ILE or transitioning to classified SLE (11, 41).

A number of SLE inception cohorts have noted the presence of organ damage by the time patients reach disease classification (42–45), and such early damage is predictive of early mortality (42, 44). Identifying early SLE signs and symptoms coupled with markers of altered immunity may be beneficial to developing a screening strategy to identify lupus relatives who would most benefit from early intervention trials compared to those who may remain in a state of latent autoimmunity without developing clinical disease. To this end, we assessed clinical, serologic, and immunological factors prior to and after SLE disease transition in two unique cohorts of lupus relatives: the lupus autoimmunity in relatives (LAUREL) follow-up cohort allowed for assessment before and after disease transition, and the lupus family registry and repository (LFRR) cohort, a confirmatory cohort assessed after the LAUREL cohort, consisting of patients with classified SLE and their blood relatives.

## 2 Materials and Methods

### 2.1 Study Population/Plasma Samples

Experiments were performed in accordance with the Helsinki Declaration and approved by the Oklahoma Medical Research Foundation (OMRF) and Medical University of South Carolina (MUSC) Institutional Review Boards (46–48). One subset of study participants were selected from the Lupus Autoimmunity in Relatives (LAUREL) follow-up cohort (11), with inclusion criteria consisting of lupus patient relatives meeting < 4 ACR SLE classification criteria (47, 48) at baseline (SLE relatives meeting ≥4 ACR criteria after medical record/serological assessment were excluded from the study) (46, 49). LAUREL cohort participants were recruited at their baseline time point from 1992-2011 and at their respective follow-up time point from 2009-2012 (**Figure S1**), an average of 6.4 years, to identify lupus relatives who transitioned to classified SLE (11). Select individuals in the LAUREL cohort were matched by sex, race, and age (± 5 years) to unaffected HC.

A confirmatory subset of study participants was selected from the Lupus Family Registry and Repository (LFRR) cohort (46), recruited from 1992-2008 (**Figure S1**), with inclusion criteria consisting of patients meeting American College of Rheumatology (ACR) classification for SLE (meeting ≥4 cumulative ACR criteria) (47, 48), relatives of SLE patients not reaching disease classification (meeting <4 ACR criteria), and unaffected healthy controls (HC). All study participants provided written informed consent along with demographic and clinical information, as well as serum and plasma samples at the time of enrollment in the LAUREL and LFRR cohorts; LAUREL cohort participants also provided serum and plasma samples at follow-up (11). Samples were stored at -20°C and assays performed on freshly thawed samples.

As outlined in the flow chart in **Figure S1**, for each nested cohort, information regarding cumulative clinical and laboratory features for each case was obtained by appropriately consented medical record review by a rheumatology-trained physician or nurse. Clinical manifestations evaluated in this protocol were determined according to criteria set by the ACR (47, 48). Stringent documentation requirements were used for review of the medical record. Each ACR criterion was recorded as being either present or absent. The date of occurrence and the presence or absence of each ACR criterion was recorded for each patient. In addition to ACR criteria, lupus relatives were assessed and scored with a modified version of the recently published SLE Risk Probability Index (mSLERPI) (50), including the following ACR criteria: malar rash, discoid rash, oral ulcers, arthritis, serositis, leukopenia, thrombocytopenia or hemolytic anemia, neurological disorder, proteinuria, ANA, and immunological disorder; alopecia, low C3 and C4, and interstitial lung disease were excluded due to insufficient data.

In addition to questionnaires to obtain demographic, education, socioeconomic, family pedigree, medical history, and medication data, participants completed the SLE-specific portion of the Connective Tissue Disease Screening Questionnaire (CSQ) (38, 40). The SLE portion of the CSQ (SLE-CSQ) was scored using an algorithm based on ACR classification criteria (38). The SLE-CSQ refers to nine criteria from the 1982 revised ACR criteria for SLE: malar rash, discoid rash, photosensitivity, oral ulcers, arthritis, serositis, proteinuria, hematologic disorder (anemia, leukopenia, low platelet count), and positive antinuclear antibody (ANA) titer. In addition, the SLE-CSQ refers to two criteria from the 1971 American Rheumatism Association criteria for SLE (alopecia and Raynaud’s phenomenon). The CSQ instrument has been validated in community-based cohorts across multiple ethnicities (38–40).

### 2.2 Detection of SLE-Associated Autoantibody Specificities

Serum samples were screened for SLE-associated autoantibodies for the purposes of determining immunologic and ANA SLE classification criteria (47, 48) in OMRF’s College of American Pathologists certified Clinical Immunology Laboratory, as previously described (51). ANAs (HEp-2 cells) and anti-double-stranded DNA (anti-dsDNA by *Crithidia luciliae*) were measured using indirect immunofluorescence (Inova Diagnostics); a positive result was defined as detection of ANAs at a titer of ≥1:120 and anti-dsDNA antibodies at a titer of ≥1:30. Precipitin levels of autoantibodies directed against Ro/SSA, La/SSB, Sm, nRNP, and ribosomal P were detected by immunodiffusion. Anticardiolipin (aCL) antibodies were measured by enzyme linked immunosorbent assay, with a titer of >10 IgG or >10 IgM units considered positive.

In addition, serum samples were screened for autoantibody specificities using the BioPlex 2200 multiplex system (Bio-Rad Technologies, Hercules, CA). The BioPlex 2200 ANA kit uses fluorescently dyed magnetic beads for simultaneous detection of 11 autoantibody specificity levels, including reactivity to dsDNA, chromatin, ribosomal P, Ro/SSA, La/SSB, Sm, the Sm/RNP complex, RNP, Scl-70, centromere B, and Jo-1, with anti-Factor XIII level serving as a control for sample integrity (51). Autoantibodies to dsDNA, chromatin, Ro/SSA, La/SSB, Sm, Sm/RNP complex, and RNP were used for analysis in the current study. Anti-dsDNA (IU/mL) has a previously determined positive cutoff of 10 IU/mL; an Antibody Index (AI) value (range 0-8) is reported by the manufacturer to reflect the fluorescence intensity of each of the other autoantibody specificities with a positive cutoff as AI=1.0. The AI scale is standardized relative to calibrators and control samples provided by the manufacturer.

### 2.3 Detection of Soluble Plasma Mediators

After verification of SLE classification criteria and status, study participants in the LAUREL cohort at follow-up and in the confirmatory LFRR nested cohort with classified SLE (≥4 cumulative ACR criteria; n=56 at follow-up in LAUREL; n=100 from LFRR), as well as lupus relatives meeting 3 ACR classification criteria (incomplete lupus, ILE; n=34 at follow-up in LAUREL; n=72 from LFRR; also verified as ILE by SLICC criteria (52)) were matched by sex and race to clinically unaffected lupus relatives (n=154 from LAUREL; n=159 from the LFRR), as well as to unaffected HC with no family history of SLE (n=77 matched to LAUREL participants; n=127 matched to LFRR participants).

Plasma levels of BLyS (R&D Systems, Minneapolis, MN) and APRIL (eBioscience/Invitrogen/ThermoFisher Scientific, Waltham, MA) were determined by enzyme-linked immunosorbent assay (ELISA), per the manufacturer protocol. An additional fifty analytes, including innate and adaptive cytokines, chemokines, and soluble TNFR superfamily members (**Table S1**), were assessed by xMAP multiplex assays (Affymetrix/eBioscience/ThermoFisher, Waltham, MA) (1, 2, 11, 18, 19).

Data were analyzed on the Bio-Rad BioPlex 200^®^ array system (Bio-Rad Technologies, Hercules, CA), with a lower boundary of 100 beads per analyte per sample. Median fluorescence intensity for each analyte was interpolated from 5-parameter logistic nonlinear regression standard curves. Analytes below the detection limit were assigned a value of 0.001 pg/mL. A known control serum was included on each plate (Cellgro human AB serum, Cat#2931949, L/N#M1016) to control for batch-effects. Well-specific validity was assessed by AssayCheX™ QC microspheres (Radix BioSolutions, Georgetown, TX, USA) to evaluate non-specific binding. Mean inter-assay coefficient of variance (CV) of multiplexed bead-based assays for cytokine detection has previously been shown to be 10-14% (53, 54) and a similar average CV (11%) was obtained across the analytes in this assay was obtained using healthy control serum. Intra-assay precision of duplicate wells averaged <10% CV in each 25-plex assay.

### 2.4 Statistical Analyses

Chi-square or Fisher’s exact test were used, as appropriate, to determine categorical differences in sex, race, and familial relationship, as well as the presence of ACR criteria, medication usage, SLE-CSQ questionnaire components, lupus-associated autoantibody specificities, and Youden index (55) determined soluble mediator positivity based on Rel vs. SLE, with Bonferroni adjusted p-values. Categorical variables significant after Bonferroni correction for multiple comparison were assessed for size effect differences, comparing odds ratios with Haldane-Anscombe correction (56). Age differences were assessed by unpaired t-test with Welch’s correction. Number of ACR criteria (ACR scores), SLE-CSQ scores, ANA titers, number of autoantibody specificities, and plasma soluble mediator levels were compared by Kruskal-Wallis test with Dunn’s multiple comparison correction. Correlations between plasma soluble mediator levels and SLE-CSQ or number of autoantibody specificities were determined by Spearman rank correlation. All statistical analyses were performed using GraphPad Prism version 9.3.1.

## 3 Results

### 3.1 Demographic and Pedigree Characteristics in Clinically Unaffected Lupus Relatives vs. Relatives With ILE or SLE

We utilized two unique and well characterized cohorts of lupus relatives to determine differences in self-reported, clinical, and serologic/immunologic features that distinguish those relatives who developed incomplete (ILE) or classified SLE vs. demographically matched, clinically unaffected lupus relatives (Rel) and unaffected healthy controls (HC). Of the 436 lupus relatives meeting <4 ACR classification criteria enrolled in the lupus autoimmunity in relatives (LAUREL) follow-up cohort at baseline, 56 (12.8%) transitioned to classified SLE and 34 (7.8%) developed ILE, meeting 3 ACR criteria at their follow-up visit, an average of 6.4 years later. These individuals were demographically matched by sex, race, and age (± 5 years) to 154 clinically unaffected Rel and 77 unaffected HC, with no demographic difference between the groups (**Table 1**) (11, 46, 49).

**Table 1:** Demographic Characteristics of Nested Lupus Relatives Study.

LAUREL^a^ Follow-up Nested Cohort	–>ILE	–>SLE	Lupus Relatives (Rel)	Unaffected HC	ILE/SLE	ILE/SLE/Rel	ILE/SLE/Rel/HC	Rel/HC
Demographics (n, %)	n=34	n=56	n=154	n=77	*p-value^b^*	*p-value^c^*	*p-value^c^*	*p-value^b^*
**Gender**					*0.4741*	*0.4654*	*0.6566*	*1.0000*
** Female**	32 (94%)	49 (88%)	142 (92%)	71 (92%)				
**Race**					*0.1645*	*0.5302*	*0.7374*	*0.7073*
** European American**	25 (74%)	43 (77%)	125 (81%)	60 (78%)				
** African American**	4 (12%)	9 (16%)	18 (12%)	9 (12%)				
** Native American**	4 (12%)	4 (7%)	8 (5%)	7 (9%)				
** Asian**	1 (2%)	0	3 (2%)	1 (1%)				
**Age (SD)**	48.9 (13.2)	47.7 (12.0)	49.3 (14.9)	52.5 (13.6)	*0.6382*	*0.7548*	*0.2161*	*0.1172*
**Multiplex Pedigree (n, %)**	9 (26%)	15 (27%)	47 (31%)	–	*1.0000*	*0.8148*	–	–
**Relationship Status (n, %)**					*0.5242*	***0.0002***	–	–
** Parent of SLE patient**	6 (18%)	10 (18%)	62 (40%)	–	*1.0000*	***0.0014***	–	–
** Child of SLE patient**	2 (6%)	10 (18%)	13 (8%)	–	*0.1239*	*0.0918*	–	–
** Sibling of SLE patient**	13 (38%)	21 (38%)	89 (58%)	–	*1.0000*	***0.0105***	–	–
** Non-FDR of SLE Patient**	9 (26%)	22 (39%)	23 (15%)	–	*0.2573*	***0.0007***	–	–
**LFRR****^a^** **Nested Cohort**	**ILE**	**SLE**	**Lupus Relatives (Rel)**	**Unaffected HC**	**ILE/SLE**	**ILE/SLE/Rel**	**ILE/SLE/Rel/HC**	**Rel/HC**
**Demographics (n, %)**	**n=72**	**n=100**	**n=159**	**n=127**	***p-value^b^***	***p-value^c^***	***p-value^c^***	***p-value^b^***
**Gender**					***0.0292***	*0.0642*	*0.1532*	*1.0000*
** Female**	68 (94%)	100 (100%)	155 (97%)	123 (97%)				
**Race**					***0.0421***	*0.0686*	*0.1374*	*0.4704*
** European American**	48 (67%)	50 (50%)	97 (61%)	72 (57%)				
** African American**	24 (33%)	50 (50%)	62 (39%)	55 (43%)				
**Age (SD)**	49.1 (13.9)	37.8 (11.3)	56.4 (14.8)	42.0 (14.7)	***<0.0001***	***<0.0001***	***<0.0001***	***<0.0001***
**Multiplex Pedigree (n, %)**	30 (42%)	20 (20%)	48 (30%)	–	***0.0036***	***0.0087***	–	–
**Relationship Status (n, %)**					*0.5279*	***<0.0001***	–	–
** Parent of SLE patient**	11 (15%)	4 (4%)	120 (75%)	–	***0.0130***	***<0.0001***	–	–
** Child of SLE patient**	3 (4%)	1 (1%)	6 (4%)	–	*0.3100*	*0.3635*	–	–
** Sibling of SLE patient**	18 (25%)	8 (8%)	42 (26%)	–	***0.0043***	***0.0010***	–	–
** Non-FDR of SLE Patient**	17 (24%)	14 (14%)	17 (11%)	–	*0.1129*	***0.0351***	–	–

a LAUREL, Lupus Autoimmunity in Relatives; LFRR, Lupus Family Registry and Repository cohort.

Categorical significance determined by ^b^Chi-square test or ^c^Fisher’s Exact test.p-values in bold are significant at **p≤0.05**.Rel, lupus relatives; HC, healthy controls; ILE, incomplete lupus erythematosus; LAUREL, Lupus Autoimmunity in Relatives; LFRR, Lupus Family Registry and Repository; SLE, systemic lupus erythematosus.

As a confirmatory cohort to the follow-up visit in the LAUREL cohort, a subset of 100 SLE patients and 72 with ILE in the LFRR were demographically matched by sex and race to 159 clinically unaffected lupus relatives and 127 unaffected HC. SLE patients in the LFRR were significantly younger (37.8 ± 11.3 years) than those in the LAUREL cohort (53.5 ± 12.0 years, *p<0.0001*). This was also true for clinically unaffected relatives (56.4 ± 14.8 years in LFRR vs. 52.5 ± 13.6 years in LAUREL, *p<0.0001*, **Table 1**).

Of interest, although the frequency of multiplex families (>1 SLE patient/family) in the LAUREL cohort was similar across ILE (26%), SLE (27%), and Rel (31%) groups (**Table 1**, *p≥0.8148*), SLE patients in the LFRR (20%) were less likely to come from multiplex families than those with ILE (42%) or clinically unaffected relatives (30%) (**Table 1**, *p≤0.0036*).

### 3.2 Lupus Type 2 Symptoms Associated With SLE-CSQ Scores and Altered BLyS and IL-10 Levels in Lupus Relatives

Recently categorized Type 2 SLE symptoms, including chronic fatigue, anxiety, depression, chronic headaches, and associated sleep disturbances are present within the context of both active and inactive SLE in patients with classified disease (33, 35). Many of these same symptoms, particularly fatigue (36, 37), often occur in the initial presentation of patients who transition to classified disease (36, 37, 57).

We evaluated baseline (prior to SLE transition) questionnaire (46) responses of self-reported chronic fatigue, anxiety, depression, chronic headaches, and hours of sleep/night (46)from lupus relatives in the nested LAUREL cohort vs. matched HC (n=77, **Table 1**). Lupus relatives were divided into those meeting no ACR criteria (No; n=61), only serologic (immunologic and ANA) ACR criteria (Ser, n=116), or clinical ACR criteria (Clin, n=67) (**Table 2**, top panel). The most consistent and significant differences were among those who reported having chronic fatigue, most frequent among lupus relatives meeting clinical ACR criteria (78%), similar among lupus relatives meeting no ACR criteria or only serologic ACR criteria (28% and 31%, respectively), yet all more frequent than matched HC (8%, p≤0.0024). Lupus relatives meeting clinical ACR criteria at baseline were also more likely to report anxiety (49%), depression (66%), chronic headaches (66%), and <7 hours of sleep/night (55%), *p≤0.0323*. Lupus relatives meeting no ACR criteria or only serologic criteria were similar to HC with respect to reporting anxiety, yet reported more chronic headaches (**Table 2**, top panel).

**Table 2:** Type 2 Symptoms in Lupus Relatives Who Transition to ILE or SLE.

LAUREL Nested Cohort	No ACR Criteria	Serologic ACR Criteria Only	Meets Clinical ACR Criteria	Unaffected HC	No/Ser/Clin^d^	No/Ser	No/Clin	Ser/Clin	No/HC	Ser/HC^d^
Baseline (Prior to SLE Transition)	n=61	n=116	n=67	n=77	*p-value* ^b^	*p-value* ^c^	*p-value* ^c^	*p-value* ^c^	*p-value* ^c^	*p-value* ^c^
**Chronic Fatigue**	17 (28%)	36 (31%)	52 (78%)	6 (8%)	***<0.0001***	*0.7314*	***<0.0001***	***<0.0001***	***0.0024***	***<0.0001***
**Anxiety**	14 (23%)	29 (25%)	33 (49%)	11 (14%)	***<0.0001***	*0.1119*	***<0.0001***	***0.0011***	*1.0000*	*0.1019*
**Depression**	21 (34%)	48 (41%)	44 (66%)	18 (23%)	***0.0006***	*0.4191*	***0.0007***	***0.0021***	*0.1840*	***0.0129***
**Chronic Headaches**	28 (46%)	52 (45%)	44 (66%)	12 (16%)	***0.0168***	*1.0000*	***0.0323***	***0.0008***	***0.0001***	***<0.0001***
**Sleep <7 hours/night****^a^**	21 (37%)	29 (26%)	35 (55%)	–	***0.0001***	***0.0019***	*0.7176*	***0.0002***	*–*	*–*
**LAUREL Nested Cohort**	**–>ILE**	**–>SLE**	**Lupus Relatives (Rel)**	**Unaffected HC**	**ILE/SLE/Rel/HC**	**ILE/SLE/Rel**	**ILE/SLE**	**ILE/Rel**	**SLE/Rel**	**Rel/HC**
**Baseline (Prior to SLE Transition)**	**n=34**	**n=56**	**n=154**	**n=77**	***p-value* ^b^**	***p-value* ^b^**	***p-value* ^c^**	***p-value* ^c^**	***p-value* ^c^**	***p-value* ^c^**
**Chronic Fatigue**	19 (56%)	46 (82%)	40 (26%)	6 (8%)	***<0.0001***	***<0.0001***	***0.0141***	***0.0018***	***<0.0001***	***0.0008***
**Anxiety**	16 (47%)	25 (45%)	35 (23%)	11 (14%)	***<0.0001***	***0.0010***	*0.8311*	***0.0057***	***0.0031***	*0.1624*
**Depression**	22 (65%)	36 (64%)	55 (36%)	18 (23%)	***<0.0001***	***<0.0001***	*1.0000*	***0.0034***	***0.0003***	*0.0715*
**Chronic Headaches**	20 (59%)	36 (64%)	68 (44%)	12 (16%)	***<0.0001***	***0.0216***	*0.6574*	*0.1326*	***0.0124***	***<0.0001***
**Sleep <7 hours/night****^a^**	14 (47%)	31 (57%)	40 (27%)	–	*–*	***0.0002***	*0.1848*	*0.0954*	***<0.0001***	*–*
**LAUREL Nested Cohort**	**ILE**	**SLE**	**Lupus Relatives (Rel)**	**Unaffected HC**	**ILE/SLE/Rel/HC**	**ILE/SLE/Rel**	**ILE/SLE**	**ILE/Rel**	**SLE/Rel**	**Rel/HC**
**Follow-up (After SLE Transition)**	**n=34**	**n=56**	**n=154**	**n=77**	***p-value* ^b^**	***p-value* ^b^**	***p-value* ^c^**	***p-value* ^c^**	***p-value* ^c^**	***p-value* ^c^**
**Chronic Fatigue**	21 (62%)	43 (77%)	43 (28%)	6 (8%)	***<0.0001***	***<0.0001***	*0.1536*	***0.0003***	***<0.0001***	***0.0003***
**Anxiety**	16 (47%)	24 (43%)	35 (23%)	11 (14%)	***<0.0001***	***0.0017***	*0.8272*	***0.0057***	***0.0055***	*0.1624*
**Depression**	18 (53%)	35 (63%)	64 (42%)	18 (23%)	***<0.0001***	***0.0223***	*0.3868*	*0.2546*	***0.0081***	***0.0084***
**Chronic Headaches**	17 (50%)	36 (64%)	47 (31%)	12 (16%)	***<0.0001***	***<0.0001***	*0.1940*	***0.0443***	***<0.0001***	***0.0162***
**Sleep <7 hours/night****^a^**	20 (67%)	26 (48%)	57 (40%)	–	*–*	***0.0223***	*0.1155*	***0.0084***	*0.3323*	*–*
**LFRR Nested Cohort**	**ILE**	**SLE**	**Lupus Relatives (Rel)**	**Unaffected HC**	**ILE/SLE/Rel/HC**	**ILE/SLE/Rel**	**ILE/SLE**	**ILE/Rel**	**SLE/Rel**	**Rel/HC**
**LFRR (After SLE Transition)**	**n=72**	**n=100**	**n=159**	**n=127**	***p-value* ^b^**	***p-value* ^b^**	***p-value* ^c^**	***p-value* ^c^**	***p-value* ^c^**	***p-value* ^c^**
**Chronic Fatigue**	55 (76%)	73 (73%)	37 (23%)	19 (14%)	***<0.0001***	***<0.0001***	*0.7237*	***<0.0001***	***<0.0001***	*0.0524*
**Anxiety**	32 (44%)	34 (34%)	33 (21%)	31 (24%)	***0.0010***	***0.0007***	*0.1646*	***0.0002***	***0.0178***	*0.4783*
**Depression**	43 (60%)	65 (65%)	50 (31%)	43 (34%)	***<0.0001***	***<0.0001***	*0.4799*	***<0.0001***	***<0.0001***	*0.7040*
**Chronic Headaches**	41 (57%)	60 (60%)	51 (32%)	39 (31%)	***<0.0001***	***<0.0001***	*0.6880*	***0.0003***	***<0.0001***	*0.8981*
**Sleep <7 hours/night****^a^**	32 (52%)	52 (52%)	60 (39%)	59 (46%)	*0.1353*	*0.0626*	*1.0000*	*0.0923*	*0.0522*	*0.2760*

a out of 33 (ILE), 53 (SLE), and 147 (Rel) reported at BL; out of 30 (ILE), 54 (SLE), and 144 (Rel) reported at FU; out of 61 (ILE), 100 (SLE), 154 (Rel), and 126 (Healthy Controls [HC]) reported in LFRR.

Categorical significance determined by ^b^Chi-square test or ^c^Fisher’s Exact test.

d **p<0.0001** No/Ser/Clin/HC all group comparisons; **p<0.0001** Clin/HC all group comparisons.p-values in bold are significant at **p<0.05**.Clin, relatives meeting clinical criteria; Rel, lupus relatives ;HC, healthy controls; LAUREL, Lupus Autoimmunity in Relatives; LFRR, Lupus Family Registry and Repository; No, relatives meeting no ACR criteria; Ser, relatives meeting only serologic criteria.

In addition, lupus relatives at baseline who transitioned to SLE at follow-up had the highest reported rate of fatigue (82%) compared to those who developed ILE (56%) or remained clinically unaffected (Rel, 26%) (**Table 2**, 2^nd^ panel, *p≤0.0141*). Yet those who transitioned to SLE at follow-up had similar frequency of reported anxiety, depression, chronic headaches, and <7 hours of sleep/night (45-64%) as those who developed ILE (47-65%), with increased frequency compared to lupus relatives who remained clinically unaffected (23-44%, **Table 2**, 3^rd^ panel, *p≤0.0124*). With the exception of anxiety and depression, where Rel had similar reported frequency as HC, lupus relatives had higher frequencies of type 2 symptoms at baseline than matched HC. This trend continued *after* transition to SLE in both the LAUREL (at follow-up) and LFRR cohorts (**Table 2**, 3^rd^ and 4^th^ panels, respectively), where SLE patients and lupus relatives with ILE had similar reported frequencies of type 2 symptoms, which were greater than clinically unaffected relatives and HC.

**Figure 1:**
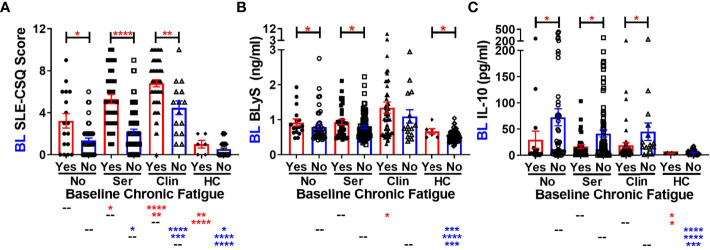
Altered SLE-CSQ scores and BLyS and IL-10 levels associated with reported chronic fatigue in lupus relatives prior to disease transition in the LAUREL cohort. Lupus relatives in the LAUREL cohort at baseline meeting No ACR criteria (No), only serologic ACR criteria (Ser), or clinical ACR criteria (Clin) vs. matched, unaffected healthy controls (HC) who did (Yes) or did not (No) report chronic fatigue on the LFRR questionnaire were evaluated for **(A)** SLE-CSQ scores, **(B)** plasma BLyS levels, and **(C)** plasma IL-10 levels. Mean ± SEM. ****p<0.0001; ***p<0.001; **p<0.01; *p<0.05 by Kruskal-Wallis with Dunn’s multiple comparison.

**Figure 2:**
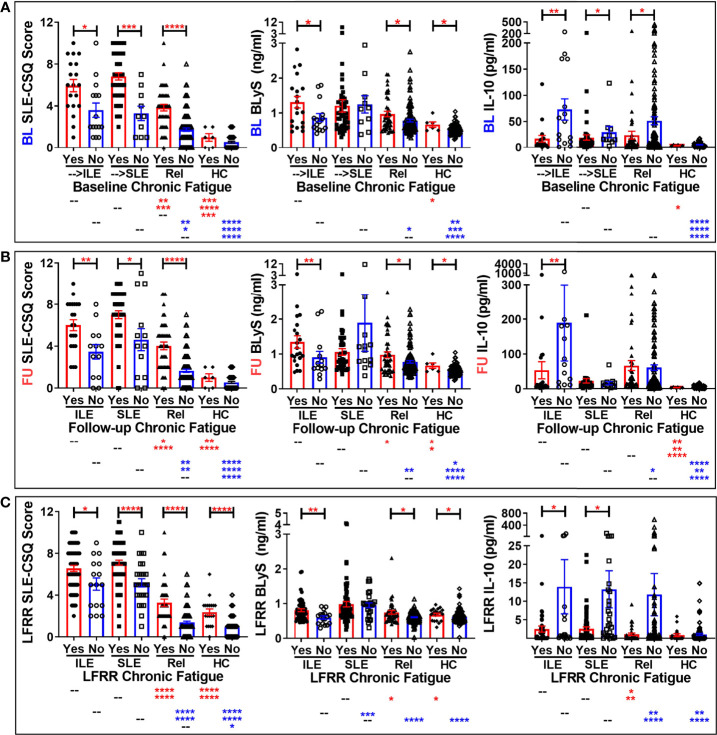
Altered SLE-CSQ scores and BLyS and IL-10 levels associated with reported chronic fatigue in lupus relatives prior to and after disease transition in the LAUREL and LFRR confirmatory cohorts. Lupus relatives who developed ILE (ILE), transitioned to SLE (SLE), or remained clinically unaffected (Rel) vs. matched, unaffected healthy controls (HC) who did (Yes) or did not (No) report chronic fatigue on the LFRR questionnaire were evaluated for SLE-CSQ scores (*1^st^ column*), plasma BLyS levels (*2^nd^ column*), and plasma IL-10 levels (*3^rd^ column*) in **(A)** LAUREL cohort at baseline (pre-transition), **(B)** LAUREL cohort at follow-up (post-transition), and **(C)** LFRR confirmatory cohort (post-transition). Mean ± SEM. ****p<0.0001; ***p<0.001; **p<0.01; *p<0.05 by Kruskal-Wallis with Dunn’s multiple comparison.

Given that lupus relatives meeting clinical ACR criteria were more likely to report type 2 symptoms, particularly fatigue, we asked if there were differences in either the SLE portion of the self-reported connective tissue disease questionnaire [SLE-CSQ; (38, 39)] or in SLE-associated immune mediators (1, 2, 11) in lupus relatives who reported fatigue at baseline in the LAUREL cohort, prior to disease transition (**Figures 1**, **2**). We observed greater SLE-CSQ scores in lupus relatives meeting no ACR criteria (No), only serologic criteria (Ser), or clinical criteria (Clin) who reported chronic fatigue (*p<0.05*, **Figure 1A**), with the highest SLE-CSQ scores, irrespective of chronic fatigue, in lupus relatives meeting clinical ACR criteria (*p<0.01*, **Figure 1A**). Of note, among the multiple serum SLE-associated autoantibody specificities and plasma immune mediators assessed, BLyS levels were *increased* in lupus relatives and HC who reported chronic fatigue, while IL-10 levels were *decreased*, irrespective of ACR criteria status (*p<0.05*, **Figures 1B, C** **)**.

We noted similar patterns of elevated SLE-CSQ scores in lupus relatives assessed by classification status who reported fatigue (**Figure 2**). Of note, BLyS levels were increased in lupus relatives who developed ILE or remained clinically unaffected and HC who reported chronic fatigue in both cohorts. However, this increase was not present in relatives who reported chronic fatigue and transitioned to SLE, either prior to disease transition (**Figure 2A**) or after reaching disease classification (**Figures 2B, C** **)**. Once again, IL-10 levels were largely decreased in lupus relatives who reported chronic fatigue in both cohorts (**Figure 2**). With respect to other type 2 symptoms, SLE-CSQ scores are likely to be increased in lupus relatives and HC who reported anxiety (**Figure S2**), depression (**Figure S3**), or chronic headaches (**Figure S4**). SLE-CSQ scores were highest in those with clinical ACR criteria prior to SLE transition (panel A), as well as those lupus relatives who transitioned to SLE, either before (panel B), or after (panels C-D) reaching SLE classification, *p<0.05*. BlyS levels were likely to be elevated in lupus relatives reporting these type 2 symptoms except those who transitioned to classified SLE, where BLyS levels were not associated with type 2 symptoms (**Figures S2–S4**). Although not necessarily significant, IL-10 levels trended higher in lupus relatives who did not report type 2 symptoms (**Figures S2–S4**). With respect to sleep (**Figure S5**), there was no consistent pattern of altered SLE-CSQ scores nor BLyS and IL-10 levels noted in either lupus relatives or HC.

### 3.3 Increased Clinical and Serologic Features Pre-Classification in Lupus Relatives Who Develop ILE or Transition to Classified SLE

**Figure 3:**
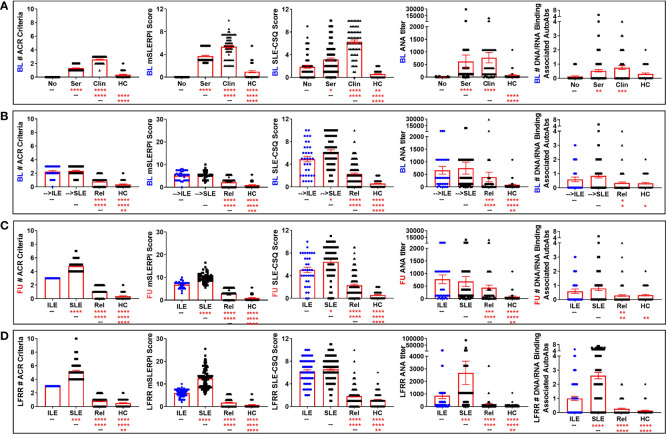
Altered ACR and SLE-CSQ scores as well as ANA titers and autoantibody accumulation in lupus relatives who develop ILE or transition to SLE. Lupus relatives and matched healthy controls (HC) were evaluated for # of ACR criteria for SLE (*1^st^ column*), modified SLE Risk Probability Index (mSLERPI) scores (*2^nd^ column*), SLE-CSQ scores (*3^rd^ column*), ANA titer (*4^th^ column*), and # of SLE-associated autoantibody specificities (*5^th^ column)* in **(A)** LAUREL cohort at baseline meeting No ACR criteria (No), only serologic ACR criteria (Ser), or clinical ACR criteria (Clin) vs. matched, unaffected HC and **(B–D)** lupus relatives who developed ILE (ILE), transitioned to SLE (SLE), or remained clinically unaffected (Rel) vs. matched healthy controls (HC) in **(B)** LAUREL cohort at baseline (pre-transition), **(C)** LAUREL cohort at follow-up (post-transition), and **(D)** LFRR confirmatory cohort (post-transition). Mean ± SEM. ****p<0.0001; ***p<0.001; **p<0.01; *p<0.05 by Kruskal-Wallis with Dunn’s multiple comparison.

In addition to Type 2 symptoms, individuals who develop ILE or transition to SLE are likely to report and/or present with serologic and/or clinical ACR criteria for SLE *prior* to disease transition (1, 2, 11, 58, 59). This may be particularly true for lupus relatives, who are at increased risk for developing SLE (9, 10, 60). At the baseline visit in the LAUREL cohort (prior to disease transition), expectedly, lupus relatives meeting clinical ACR criteria had higher ACR scores (number of ACR criteria) and modified SLE Risk Probability Index (mSLERPI) (50) scores than those meeting only serologic criteria (*p<0.0001*, **Figure 3A**, 1^st^ and 2^nd^ columns, respectively). Of interest, those relatives who were destined to develop ILE or transition to SLE at follow-up met a similar number of ACR and mSLERPI criteria at baseline (**Figure 3B**, 1^st^ and 2^nd^ columns, respectively). This is reflective of the lack of significant difference in the clinical and serologic (immunologic and ANA) ACR criteria met at baseline, as well as frequency of immune modulating treatments, in the LAUREL cohort for those relatives who developed ILE or transitioned to SLE at follow-up (**Table 3**). However, despite the lack of significance (*p≥0.2390*), it was noted that only those relatives who transitioned to SLE at follow-up presented with serositis (n=4, 7%) or neurologic (n=1, 2%) criteria at baseline. Also of note, relatives who remained clinically unaffected, or met only serologic criteria at baseline, had higher baseline ACR scores than matched HC, likely due to the higher rate of ANA positivity (IIF titer ≥1:120) in clinically unaffected relatives (51%) vs. HC (18%), both of which were significantly lower than those who developed ILE (88%) or transitioned to SLE (91%) (*p<0.0001*, **Table 3**).

**Table 3:** ACR Criteria and Medication in LAUREL Nested Cohort at Baseline (Prior to SLE Transition).

	–>ILE	–>SLE	Lupus Relatives (Rel)	Unaffected HC	ILE/SLE	ILE/SLE/Rel	ILE/SLE/Rel/HC	Rel/HC
ACR Classification Criteria (n,%)	n=34	n=56	n=154	n=77	*p-value^b^*	*p-value^c^*	*p-value^c^*	*p-value^b^*
**Malar Rash**	3 (8%)	7 (13%)	–	–	*0.7368*	*–*	*–*	*–*
**Discoid Rash**	1 (3%)	1 (2%)	–	–	*1.0000*	*–*	*–*	*–*
**Photosensitivity**	7 (21%)	14 (25%)	–	–	*0.7981*	*–*	*–*	*–*
**Oral Ulcers**	1 (3%)	2 (4%)	–	–	*1.0000*	*–*	*–*	*–*
**Arthritis**	10 (29%)	20 (36%)	–	–	*0.6465*	*–*	*–*	*–*
**Serositis**	0	4 (7%)	–	–	*0.2930*	*–*	*–*	*–*
** Pericarditis**	0	1 (2%)	–	–	*1.0000*	*–*	*–*	*–*
** Pleuritis**	0	3 (5%)	–	–	*0.2689*	*–*	*–*	*–*
**Renal**	1 (3%)	1 (2%)	–	–	*1.0000*	*–*	*–*	*–*
** Proteinuria**	1 (3%)	1 (2%)	–	–	*1.0000*	*–*	*–*	*–*
** Cellular Casts**	0	0	–	–	*1.0000*	*–*	*–*	*–*
**Neurologic**	0	1 (2%)	–	–	*1.0000*	*–*	*–*	*–*
** Seizure**	0	1 (2%)	–	–	*1.0000*	*–*	*–*	*–*
** Psychosis**	0	0	–	–	*1.0000*	*–*	*–*	*–*
**Hematologic**	6 (18%)	5 (9%)	–	–	*0.3200*	*–*	*–*	*–*
** Hemolytic Anemia**	0	0	–	–	*1.0000*	*–*	*–*	*–*
** Thrombocytopenia**	0	0	–	–	*1.0000*	*–*	*–*	*–*
** Leukopenia**	4 (12%)	3 (5%)	–	–	*0.4260*	*–*	*–*	*–*
** Lymphopenia**	4 (12%)	4 (7%)	–	–	*0.7070*	*–*	*–*	*–*
**Immunologic** ^**a**^	17 (50%)	25 (45%)	41 (27%)	14 (18%)	*0.8241*	***0.0004***	***<0.0001***	*0.1904*
** anti-dsDNA**	5 (15%)	6 (11%)	1 (1%)	0	*0.7415*	***0.0002***	***<0.0001***	*1.0000*
** anti-Sm**	0	1 (2%)	0	0	*1.0000*	*–*	*–*	*–*
** anti-cardiolipin (aCL)**	14 (41%)	18 (32%)	40 (26%)	14 (18%)	*0.4962*	*0.1885*	*0.0611*	*0.2481*
**ANA**	30 (88%)	51 (91%)	78 (51%)	14 (18%)	*0.7249*	***<0.0001***	***<0.0001***	***<0.0001***
**Medications (n, %)**	**n=34**	**n=56**	**n=154**	**n=77**	***p-value^b^***	***p-value^c^***	***p-value^c^***	***p-value^b^***
**Steroid**	15 (44%)	33 (59%)	6 (4%)	1 (1%)	*0.1963*	***<0.0001***	***<0.0001***	*0.4292*
**Hydroxychloroquine**	16 (47%)	34 (61%)	5 (3%)	0	*0.2744*	***<0.0001***	***<0.0001***	*0.1723*
**Immunosuppressant****^d^**	6 (18%)	14 (25%)	1 (1%)	0	*0.4485*	***<0.0001***	***<0.0001***	*1.0000*
**Major Immunosuppressant****^d^**	1 (3%)	4 (7%)	0	0	*0.6462*	***0.0326***	***0.0072***	*1.0000*
**Biologic**	0	0	0	0	*–*	*–*	*–*	*–*

a Seropositivity determined by Crithidia luciliae assay (anti-dsDNA; titer≥1:30), gel precipitation assay (anti-Sm), or ELISA (aCL; >10 IgG or IgM units).

Categorical significance determined by ^b^Chi-square test or ^c^Fisher’s Exact test.

d Immunosuppressant = methotrexate, azathioprine; Major Immunosuppressant = mycophenolate mofetil, cyclophosphamide.p-values in bold are significant at **p≤0.05**. ANA, antinuclear antibodies; Rel, lupus relatives; HC, healthy controls; ILE, incomplete lupus erythematosus; SLE, systemic lupus erythemtosus.

At the follow-up time point (post-SLE transition) in the LAUREL cohort, those relatives who had ILE had similar frequency of accumulated hematologic and serologic (immunologic/ANA) criteria as those who transitioned to SLE, while those with classified SLE had accumulated a higher frequency of mucocutaneous (malar rash, discoid rash, photosensitivity, and oral ulcers), arthritis, serositis, and neurologic criteria (*p≤0.0273*, **Table 4**). This was reflective of both the expected increase in number of ACR and mSLERPI criteria (*p<0.0001*, **Figure 3C**, 1^st^ and 2^nd^ columns, respectively) and increase in hydroxychloroquine use (*p=0.0051*, **Table 4**), but not other immune modulating treatments, in those lupus relatives who transitioned to classified SLE compared to those relatives with ILE at follow-up. While relatives who remained clinically unaffected also had lower rates of meeting immunologic criteria (36%) or being ANA positive (64%) compared to relatives who developed ILE (62% and 97%, respectively) or transitioned to SLE (55% and 96%, respectively) at follow-up in the LAUREL cohort (*p≤0.0451*, **Table 4**), they were also significantly higher than matched, unaffected HC, with 18% frequency in meeting immunologic criteria and ANA positivity (*p≤0.0061*, **Table 4**).

**Table 4:** ACR Criteria and Medication in LAUREL Nested Cohort at Follow-up (After SLE Transition).

	ILE	SLE	Lupus Relatives (Rel)	Unaffected HC	ILE/SLE	ILE/SLE/Rel	ILE/SLE/Rel/HC	Rel/HC
ACR Classification Criteria (n,%)	n=34	n=56	n=154	n=77	*p-value^b^*	*p-value^c^*	*p-value^c^*	*p-value^b^*
**Malar Rash**	5 (15%)	33 (59%)	–	–	***<0.0001***	*–*	*–*	*–*
**Discoid Rash**	1 (3%)	10 (18%)	–	–	***0.0469***	*–*	*–*	*–*
**Photosensitivity**	9 (26%)	29 (52%)	–	–	***0.0273***	*–*	*–*	*–*
**Oral Ulcers**	5 (15%)	25 (45%)	–	–	***0.0052***	*–*	*–*	*–*
**Arthritis**	19 (56%)	42 (75%)	–	–	*0.0677*	*–*	*–*	*–*
**Serositis**	0	25 (45%)	–	–	***<0.0001***	*–*	*–*	*–*
** Pericarditis**	0	7 (13%)	–	–	***0.0418***	*–*	*–*	*–*
** Pleuritis**	0	23 (41%)	–	–	***<0.0001***	*–*	*–*	*–*
**Renal**	1 (3%)	5 (9%)	–	–	*0.4026*	*–*	*–*	*–*
** Proteinuria**	1 (3%)	5 (9%)	–	–	*0.4026*	*–*	*–*	*–*
** Cellular Casts**	0	0	–	–	*–*	*–*	*–*	*–*
**Neurologic**	0	7 (13%)	–	–	***0.0418***	*–*	*–*	*–*
** Seizure**	0	5 (9%)	–	–	*0.1523*	*–*	*–*	*–*
** Psychosis**	0	2 (4%)	–	–	*0.5246*	*–*	*–*	*–*
**Hematologic**	8 (24%)	8 (14%)	–	–	*0.2734*	*–*	*–*	*–*
** Hemolytic Anemia**	1 (3%)	1 (2%)	–	–	*1.0000*	*–*	*–*	*–*
** Thrombocytopenia**	1 (3%)	0	–	–	*0.3778*	*–*	*–*	*–*
** Leukopenia**	5 (15%)	5 (9%)	–	–	*0.4942*	*–*	*–*	*–*
** Lymphopenia**	5 (15%)	4 (7%)	–	–	*0.2899*	*–*	*–*	*–*
**Immunologic****^a^**	21 (62%)	31 (55%)	55 (36%)	14 (18%)	*0.6810*	***0.0031***	***<0.0001***	***0.0061***
** anti-dsDNA**	6 (18%)	9 (16%)	1 (1%)	0	*1.0000*	***<0.0001***	***<0.0001***	*1.0000*
** anti-Sm**	0	2 (4%)	0	0	*0.5246*	*–*	*–*	*–*
** anti-cardiolipin (aCL)**	13 (38%)	14 (25%)	29 (19%)	14 (18%)	*0.2437*	***0.0451***	*0.0636*	*1.0000*
**ANA**	33 (97%)	54 (96%)	98 (64%)	14 (18%)	*1.0000*	***<0.0001***	***<0.0001***	***<0.0001***
**Medications (n, %)**	**n=34**	**n=56**	**n=154**	**n=77**	***p-value^b^***	***p-value^c^***	***p-value^c^***	***p-value^b^***
**Steroid**	17 (50%)	21 (38%)	7 (5%)	1 (1%)	*0.0861*	***<0.0001***	***<0.0001***	*0.2745*
**Hydroxychloroquine**	11 (32%)	35 (64%)	8 (5%)	0	***0.0051***	***<0.0001***	***<0.0001***	*0.0546*
**Immunosuppressant****^d^**	13 (38%)	17 (30%)	5 (3%)	0	*0.4935*	***<0.0001***	***<0.0001***	*0.1723*
**Major Immunosuppressant****^d^**	1 (3%)	4 (7%)	1 (1%)	0	*0.6462*	***0.0265***	***0.0097***	*1.0000*
**Biologic**	2 (6%)	0	0	0	*0.1401*	***0.0020***	***0.0007***	*1.0000*

a Seropositivity determined by Crithidia luciliae assay (anti-dsDNA; titer≥1:30), gel precipitation assay (anti-Sm), or ELISA (aCL; >10 IgG or IgM units).

Categorical significance determined by ^b^Chi-square test or ^c^Fisher’s Exact test.

d Immunosuppressant = methotrexate, azathioprine; Major Immunosuppressant = mycophenolate mofetil, cyclophosphamide.p-values in bold are significant at **p≤0.05**.ANA, antinuclear antibodies; Rel, lupus relatives; HC, healthy controls; ILE, incomplete lupus erythematosus; SLE, systemic lupus erythemtosus.

We wanted to know if lupus relatives with classified SLE or ILE, as well as clinically unaffected relatives and matched HC in the confirmatory LFRR nested cohort had a similar profile of ACR criteria as those at follow-up in the LAUREL cohort. The number of ACR and mSLERPI criteria met in the lupus relative groups and HC were similar between the LFRR (**Figure 3D**, 1^st^ and 2^nd^ columns, respectively) and follow-up, post-SLE transition visit in the LAUREL cohort (**Figure 3C**, 1^st^ and 2^nd^ columns), including increased ACR and mSLERPI scores in clinically unaffected relatives vs. HC (*p<0.01*). However, relatives with classified SLE in the confirmatory LFRR nested cohort had a greater frequency of renal (59% vs. 9% in LAUREL, *p<0.0001*), hematologic (54% vs. 14%, *p<0.0001*), and immunologic (94% vs. 55%, *p<0.0001*) ACR criteria (**Tables 5**, **6**). In contrast, relatives who transitioned to SLE in LAUREL at follow-up were more likely to meet mucocutaneous ACR criteria, including malar rash (59% vs. 35% in LFRR, *p=0.0044*), photosensitivity (52% vs. 35%, p=0.0440), oral ulcers (45% vs. 25%, *p=0.0195*).

**Table 5:** ACR Criteria and Medication in LFRR Confirmatory Nested Cohort (After SLE Transition).

	ILE	SLE	Lupus Relatives (Rel)	Unaffected HC	ILE/SLE	ILE/SLE/Rel	ILE/SLE/Rel/HC	Rel/HC
ACR Classification Criteria (n,%)	n=72	n=100	n=159	n=127	*p-value^b^*	*p-value^c^*	*p-value^c^*	*p-value^b^*
**Malar Rash**	13 (18%)	35 (35%)	–	–	***0.0162***	*–*	*–*	*–*
**Discoid Rash**	6 (8%)	12 (12%)	–	–	*0.6146*	*–*	*–*	*–*
**Photosensitivity**	26 (36%)	35 (35%)	–	–	*1.0000*	*–*	*–*	*–*
**Oral Ulcers**	5 (7%)	25 (25%)	–	–	***0.0020***	*–*	*–*	*–*
**Arthritis**	27 (38%)	68 (68%)	–	–	***<0.0001***	*–*	*–*	*–*
**Serositis**	7 (10%)	37 (37%)	–	–	***<0.0001***	*–*	*–*	*–*
** Pericarditis**	4 (6%)	17 (17%)	–	–	***0.0322***	*–*	*–*	*–*
** Pleuritis**	4 (6%)	28 (28%)	–	–	***0.0001***	*–*	*–*	*–*
**Renal**	2 (3%)	49 (49%)	–	–	***<0.0001***	*–*	*–*	*–*
** Proteinuria**	2 (3%)	48 (48%)	–	–	***<0.0001***	*–*	*–*	*–*
** Cellular Casts**	0	20 (20%)	–	–	***<0.0001***	*–*	*–*	*–*
**Neurologic**	1 (1%)	13 (13%)	–	–	***0.0085***	*–*	*–*	*–*
** Seizure**	0	8 (8%)	–	–	***0.0214***	*–*	*–*	*–*
** Psychosis**	1 (1%)	5 (5%)	–	–	*0.4027*	*–*	*–*	*–*
**Hematologic**	25 (35%)	54 (54%)	–	–	*0.5829*	*–*	*–*	*–*
** Hemolytic Anemia**	0	7 (7%)	–	–	***0.0424***	*–*	*–*	*–*
** Thrombocytopenia**	2 (3%)	20 (20%)	–	–	***0.0008***	*–*	*–*	*–*
** Leukopenia**	16 (22%)	30 (30%)	–	–	*0.2969*	*–*	*–*	*–*
** Lymphopenia**	11 (15%)	31 (31%)	–	–	***0.0198***	*–*	*–*	*–*
**Immunologic****^a^**	34 (47%)	94 (94%)	59 (37%)	38 (30%)	***<0.0001***	***<0.0001***	***<0.0001***	*0.2114*
** anti-dsDNA**	6 (8%)	75 (75%)	1 (1%)	0	***<0.0001***	***<0.0001***	***<0.0001***	*1.0000*
** anti-Sm**	1 (1%)	33 (33%)	0	0	***<0.0001***	*–*	*–*	*–*
** anti-cardiolipin (aCL)**	31 (43%)	63 (63%)	59 (37%)	38 (30%)	***0.0129***	***0.0002***	***<0.0001***	*0.2114*
**ANA**	67 (93%)	91 (91%)	69 (43%)	27 (21%)	*0.7799*	***<0.0001***	***<0.0001***	***<0.0001***
**Medications (n, %)**	**n=66**	**n=100**	**n=135**	**n=100**	***p-value^b^***	***p-value^c^***	***p-value^c^***	***p-value^b^***
**Steroid**	51 (77%)	94 (94%)	5 (4%)	7 (7%)	***0.0033***	***<0.0001***	***<0.0001***	*0.3695*
**Hydroxychloroquine**	40 (61%)	86 (86%)	1 (1%)	0	***0.0003***	***<0.0001***	***<0.0001***	*1.0000*
**Immunosuppressant****^d^**	14 (21%)	55 (55%)	1 (1%)	1 (1%)	***<0.0001***	***<0.0001***	***<0.0001***	*1.0000*
**Major Immunosuppressant****^d^**	5 (8%)	51 (51%)	0	0	***<0.0001***	***<0.0001***	***<0.0001***	*–*
**Biologic**	0	4 (4%)	0	0	*0.1522*	***0.0142***	***0.0030***	*–*

a Seropositivity determined by Crithidia luciliae assay (anti-dsDNA; titer≥1:30), gel precipitation assay (anti-Sm), or ELISA (aCL; >10 IgG or IgM units).

Categorical significance determined by ^b^Chi-square test or ^c^Fisher’s Exact test.

d Immunosuppressant = methotrexate, azathioprine; Major Immunosuppressant = mycophenolate mofetil, cyclophosphamide.p-values in bold are significant at **p≤0.05**. ANA, antinuclear antibodies; Rel, lupus relatives; HC, healthy controls; ILE, incomplete lupus erythematosus; SLE, systemic lupus erythemtosus.

**Table 6:** SLE-CSQ Components in Lupus Relatives Who Transition to ILE or SLE.

LAUREL Nested Cohort	–>ILE	–>SLE	Lupus Relatives (Rel)	Unaffected HC	ILE/SLE	ILE/SLE/Rel	ILE/SLE/Rel/HC	Rel/HC
Baseline (Prior to SLE Transition)	n=34	n=56	n=154	n=77	*p-value^a^*	*p-value^b^*	*p-value^b^*	*p-value^a^*
**Cheek rash**	10 (29%)	29 (52%)	13 (8%)	1 (1%)	***0.0134***	***<0.0001***	***<0.0001***	***0.0387***
**Discoid lupus**	2 (6%)	0	0	0	*0.1401*	***0.0020***	***0.0007***	*1.0000*
**Sun sensitivity**	15 (44%)	35 (63%)	42 (27%)	1 (1%)	*0.1255*	***<0.0001***	***<0.0001***	***<0.0001***
**Mouth sores**	9 (26%)	35 (63%)	32 (21%)	3 (4%)	***0.0011***	***<0.0001***	***<0.0001***	***0.0004***
**Arthritis**	23 (68%)	43 (77%)	62 (40%)	13 (17%)	*0.4613*	***<0.0001***	***<0.0001***	***<0.0001***
**Pleurisy**	12 (35%)	33 (59%)	35 (23%)	2 (3%)	***0.0496***	***<0.0001***	***<0.0001***	***<0.0001***
**Protein in urine**	16 (47%)	26 (46%)	21 (14%)	2 (3%)	*1.0000*	***<0.0001***	***<0.0001***	***0.0089***
**Seizure**	6 (18%)	7 (13%)	5 (3%)	0	*0.5457*	***0.0036***	***0.0001***	*0.1723*
**Low blood counts**	26 (76%)	39 (70%)	62 (40%)	17 (22%)	*0.6284*	***<0.0001***	***<0.0001***	***0.0078***
**Positive ANA**	19 (56%)	38 (68%)	20 (13%)	0	*0.4895*	***<0.0001***	***<0.0001***	***0.0003***
**Cold sensitivity**	14 (41%)	32 (57%)	37 (24%)	6 (8%)	*0.1923*	***<0.0001***	***<0.0001***	***0.0023***
**Rapid hair loss**	13 (38%)	30 (54%)	24 (16%)	1 (1%)	*0.1941*	***<0.0001***	***<0.0001***	***0.0005***
**LAUREL Nested Cohort**	**ILE**	**SLE**	**Lupus Relatives (Rel)**	**Unaffected HC**	**ILE/** **SLE**	**ILE/SLE/** **Rel**	**ILE/SLE/** **Rel/HC**	**Rel/HC**
**Follow-up (After SLE Transition)**	**n=34**	**n=56**	**n=154**	**n=77**	***p-value^a^***	***p-value^b^***	***p-value^b^***	***p-value^a^***
**Cheek rash**	10 (29%)	28 (50%)	14 (9%)	1 (1%)	*0.0781*	***<0.0001***	***<0.0001***	***0.0233***
**Discoid lupus**	5 (15%)	4 (7%)	3 (2%)	0	*0.2899*	***0.0053***	***0.0005***	*0.5526*
**Sun sensitivity**	18 (53%)	41 (73%)	35 (23%)	1 (1%)	*0.0675*	***<0.0001***	***<0.0001***	***<0.0001***
**Mouth sores**	11 (32%)	37 (66%)	33 (21%)	3 (4%)	***0.0024***	***<0.0001***	***<0.0001***	***0.0004***
**Arthritis**	26 (76%)	44 (79%)	66 (43%)	13 (17%)	*0.8006*	***<0.0001***	***<0.0001***	***<0.0001***
**Pleurisy**	12 (35%)	31 (55%)	27 (18%)	2 (3%)	*0.0829*	***<0.0001***	***<0.0001***	***0.0006***
**Protein in urine**	10 (29%)	30 (54%)	18 (12%)	2 (3%)	***0.0302***	***<0.0001***	***<0.0001***	***0.0238***
**Seizure**	4 (12%)	8 (14%)	6 (4%)	0	*1.0000*	***0.0224***	***0.0012***	*0.1822*
**Low blood counts**	22 (65%)	38 (68%)	55 (36%)	17 (22%)	*0.8195*	***<0.0001***	***<0.0001***	***0.0362***
**Positive ANA**	25 (74%)	48 (86%)	32 (21%)	0	*0.1734*	***<0.0001***	***<0.0001***	***<0.0001***
**Cold sensitivity**	16 (47%)	32 (57%)	43 (28%)	6 (8%)	*0.3894*	***0.0002***	***<0.0001***	***0.0003***
**Rapid hair loss**	11 (32%)	29 (52%)	25 (16%)	1 (1%)	*0.0838*	***<0.0001***	***<0.0001***	***0.0003***
**LFRR Nested Cohort**	**ILE**	**SLE**	**Lupus Relatives (Rel)**	**Unaffected HC**	**ILE/** **SLE**	**ILE/SLE/** **Rel**	**ILE/SLE/** **Rel/HC**	**Rel/HC**
**Follow-up (After SLE Transition)**	**n=72**	**n=100**	**n=159**	**n=127**	***p-value^a^***	***p-value^b^***	***p-value^b^***	***p-value^a^***
**Cheek rash**	39 (54%)	57 (57%)	10 (7%)	3 (2%)	*0.7568*	***<0.0001***	***<0.0001***	*0.1520*
**Discoid lupus**	0	0	0	0	–	–	–	–
**Sun sensitivity**	47 (65%)	59 (59%)	26 (16%)	8 (6%)	*0.4305*	***<0.0001***	***<0.0001***	***0.0098***
**Mouth sores**	38 (53%)	50 (50%)	13 (8%)	10 (8%)	*0.7586*	***<0.0001***	***<0.0001***	*1.0000*
**Arthritis**	58 (81%)	73 (73%)	72 (45%)	29 (23%)	*0.3724*	***<0.0001***	***<0.0001***	***0.0001***
**Pleurisy**	44 (61%)	57 (57%)	25 (9%)	15 (12%)	*0.6393*	***<0.0001***	***<0.0001***	*0.5631*
**Protein in urine**	31 (43%)	76 (76%)	21 (13%)	8 (6%)	***<0.0001***	***<0.0001***	***<0.0001***	*0.0748*
**Seizure**	8 (11%)	25 (25%)	7 (4%)	4 (3%)	***0.0301***	***<0.0001***	***<0.0001***	*0.7598*
**Low blood counts**	51 (71%)	89 (89%)	59 (37%)	42 (33%)	***0.0081***	***<0.0001***	***<0.0001***	*0.5341*
**Positive ANA**	46 (64%)	68 (68%)	11 (7%)	2 (2%)	*0.6252*	***<0.0001***	***<0.0001***	***0.0431***
**Cold sensitivity**	47 (65%)	60 (60%)	22 (14%)	13 (10%)	***0.0422***	***<0.0001***	***<0.0001***	*0.3416*
**Rapid hair loss**	33 (46%)	48 (48%)	17 (11%)	8 (6%)	*0.8771*	***<0.0001***	***<0.0001***	*0.2126*

Categorical significance determined by ^a^Chi-square test or ^b^Fisher’s Exact test.p-values in bold are significant at **p≤0.05**.Rel, lupus relatives; HC, healthy controls; ILE, incomplete lupus erythematosus; SLE, systemic lupus erythemtosus.

Arthritis, serositis, and neurologic clinical criteria, as well as rate of ANA positivity, were similar between relatives with classified SLE in the LFRR (13-68%) vs. LAUREL (13-75%) follow-up cohorts (**Tables 4**, **5**). Similar to the LAUREL cohort, SLE patients (12-86%) in the LFRR cohort were more likely than relatives with ILE (8-61%) to meet mucocutaneous, serositis, and neurologic ACR criteria, as well as be prescribed hydroxychloroquine. However, SLE patients (49-94%) in the LFRR cohort were also more likely than their counterparts with ILE (3-47%) to meet arthritis, renal, and immunologic criteria (*p<0.0001*, **Table 5**), reflected with increased rates of immune modulating treatments, including steroids (94% SLE vs. 77% ILE, *p=0.0033*, **Table 5**). Clinically unaffected relatives (1-37%) in the LFRR had similar rates of immunologic criteria and immune modulating treatments as matched HC (1-30%), but were once again more likely than HC to be ANA positive (43% Rel vs. 21% HC, *p<0.0001*, **Table 5**), reinforcing an important difference between lupus relatives who remain clinically unaffected and demographically matched healthy individuals in the general population.

### 3.4 Participant-Reported SLE-CSQ Increased in Lupus Relatives and Reflects Future SLE Classification Status

ACR scores for SLE classification reflect a cumulative combination of currently observed and previously documented clinical and serologic criteria (47). The SLE portion of the CSQ is based on the ACR classification criteria for SLE and may serve as a useful screening tool for identifying individuals at risk of developing SLE (11, 34, 38–41). Although validated only in the general population (38, 40), we sought to determine if the SLE-CSQ scores and reported symptoms were reflective of medical record confirmed SLE classification status in lupus relatives. At the baseline visit in the LAUREL cohort, we noted that lupus relatives had significantly higher SLE-CSQ scores than matched HC (**Figure 3A**, 3^rd^ column), with the highest scores in relatives meeting clinical ACR criteria (*p<0.0001*), followed by serologic criteria only (*p<0.0001*) and no classification criteria (*p=0.0021*). Relatives who would transition to SLE at follow-up had higher SLE-CSQ scores than those who will develop ILE (*p=0.0354*, **Figure 3B**, 3^rd^ column). Post-transition, relatives with classified SLE continued to have higher SLE-CSQ scores than those with ILE (*p=0.0142*, **Figure 3C**, 3^rd^ column) in the LAUREL cohort, while relatives with classified SLE in the LFRR cohort had similar SLE-CSQ scores in the LFRR cohort (**Figure 3D**, 3^rd^ column).

Of note, clinically unaffected relatives in both the LAUREL (baseline and follow-up) and LFRR confirmatory cohorts had lower SLE-CSQ scores than those who developed ILE or transitioned to SLE (*p<0.0001*), yet significantly higher than unaffected HC (*p<0.0001*, **Figures 3B–D**, 3^rd^ column). This was also true across the individual component responses, where clinically unaffected relatives were less likely to note individual symptoms than their SLE and ILE counterparts (*p<0.05*) in both LAUREL (baseline and follow-up) and LFRR cohorts (**Table 6**), yet more likely than matched, unaffected HC to report symptoms, particularly sun sensitivity (*p≤0.0098*), pleurisy (*p≤0.0001*), and positive ANA (*p≤0.0431*). Lupus relatives who transitioned to SLE were more likely than those who developed ILE to report cheek rash (*p=0.0134*), mouth sores (*p=0.0011*), and pleurisy (*p=0.0496*) at baseline (LAUREL), mouth sores and protein in the urine at follow-up (LAUREL), and protein in the urine, seizure, and low blood counts (LFRR). In contrast, relatives with ILE in the LFRR cohort were more likely to report cold sensitivity (*p=0.0422*, **Table 6**)

**Figure 4:**
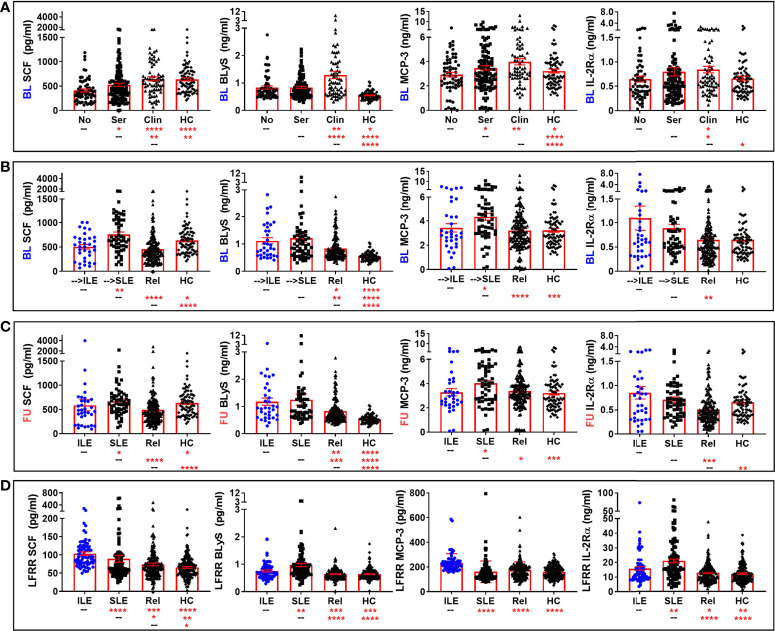
Altered pro-inflammatory mediators in lupus relatives who develop ILE or transition to SLE. Lupus relatives and matched healthy controls (HC) were evaluated for plasma levels of stem cell factor (SCF; *1^st^ column*), BLyS (*2^nd^ column*), MCP-3 (*3^rd^ column*), and soluble IL-2Rα (*4^th^ column*) in **(A)** LAUREL cohort at baseline meeting No ACR criteria (No), only serologic ACR criteria (Ser), or clinical ACR criteria (Clin) vs. matched, unaffected HC and **(B-D)** lupus relatives who developed ILE (ILE), transitioned to SLE (SLE), or remained clinically unaffected (Rel) vs. matched, unaffected healthy controls (HC) in **(B)** LAUREL cohort at baseline (pre-transition), **(C)** LAUREL cohort at follow-up (post-transition), and **(D)** LFRR confirmatory cohort (post-transition). Mean ± SEM. ****p<0.0001; ***p<0.001; **p<0.01; *p<0.05 by Kruskal-Wallis with Dunn’s multiple comparison.

Overall, SLE-CSQ scores closely correlated with the number of ACR criteria documented in the medical record across the LAUREL (baseline and follow-up) and LFRR cohorts (Spearman r≥0.526 [0.426-0.614 95% CI], *p<0.0001*, **Table 7**), as well as ANA titer (Spearman r≥0.238 [0.113-0.367], *p=0.0002*, **Table 7**) and number of autoantibody specificities (Spearman r≥0.140 [0.011-0.265], *p=0.0286*, **Table 7**). The number of autoantibody specificities detected in both the LAUREL (baseline and follow-up) and LFRR cohorts also correlated with number of ACR criteria documented in the medical record (Spearman r≥0.238 [0.113-0.357], *p≤0.0002*, **Table 7**) and ANA titers (Spearman r≥0.313 [0.191-0.425], *p<0.0001*, **Table 7**). Lupus relatives meeting clinical criteria at baseline in the LAUREL cohort had similar ANA titers and number of SLE-associated autoantibody specificities as those meeting only serologic criteria, yet higher (*p<0.0001*) than matched relatives with no ACR criteria and unrelated HC, which had similar profiles (**Figure 3A**, 4^th^-5^th^ columns). This was also true when comparing relatives who developed ILE or transitioned to SLE, with similar ANA titers and number of SLE-associated autoantibody specificities at baseline and follow-up in the LAUREL cohort that were higher (*p<0.001*) than matched, clinically unaffected relatives and unaffected HC (**Figures 3B, C**, 4^th^-5^th^ columns).

**Table 7:** Correlation Between SLE-CSQ Score, ACR Score, and SLE-Associated Autoantibody Specificities in Lupus Relatives.

SLE-SCQ Score vs.	LAUREL (BL) Nested Cohort			LAUREL (FU) Nested Cohort			LFRR Nested Cohort		
	Spearman r	95% CI	*p-value^a^*	Spearman r	95% CI	*p-value^a^*	Spearman r	95% CI	*p-value^a^*
**ACR Score**	0.526	0.426 to 0.614	***<0.0001***	0.562	0.467 to 0.645	***<0.0001***	0.710	0.650 to 0.761	***<0.0001***
**ANA titer**	0.328	0.208 to 0.439	***<0.0001***	0.238	0.113 to 0.357	***0.0002***	0.428	0.332 to 0.514	***<0.0001***
**# of SLE-associated AutoAbs**	0.190	0.062 to 0.311	***0.0029***	0.140	0.011 to 0.265	***0.0286***	0.340	0.237 to 0.434	***<0.0001***
**# SLE-associated AutoAbs vs.**	**Spearman r**	**95% CI**	***p-value^a^***	**Spearman r**	**95% CI**	***p-value^a^***	**Spearman r**	**95% CI**	***p-value^a^***
**# ACR Criteria**	0.238	0.113 to 0.357	***0.0002***	0.296	0.173 to 0.409	***<0.0001***	0.525	0.440 to 0.601	***<0.0001***
**ANA titer**	0.313	0.191 to 0.425	***<0.0001***	0.376	0.259 to 0.482	***<0.0001***	0.561	0.480 to 0.633	***<0.0001***

a Spearman correlation Bonferroni corrected **p≤0.0017**.All p-values **≤0.05** in **bold**. All p-values **≤0.0017 bold and underlined** to denote continued significance with Bonferonni correction.ANA, antinuclear antibodies; BL, baseline; FU, follow-up; ACR, American College of Rheumatology; AutoAbs, autoantibodies.

However, relatives with classified SLE in the confirmatory LFRR cohort had the highest ANA titers and number of SLE-associated autoantibody specificities, followed by relatives who developed ILE, clinically unaffected relatives, and matched HC, with significant differentiation between the groups (*p<0.01*, **Figure 3D**, 4^th^-5^th^ columns). This was associated with an increased likelihood of LFRR SLE patients to be positive for autoantibody specificities to dsDNA (44%, *p<0.0001*), chromatin (49%, *p≤0.0002*), and nucleosome antigens, including Sm (35%, *p<0.0001*), SmRNP (43%, *p≤0.0001*), and RNP (41%, *p≤0.0003*) compared to relatives with ILE (1-21%), clinically unaffected relatives (1-9%), and unaffected HC (0-3%, **Table 8**). In contrast, relatives who transitioned to SLE had similar rates of autoantibody positivity to Ro/SSA (25-38%) and La/SSB (11-12%) compared to those with ILE (24-26%, Ro/SSA; 15%, La/SSB) in both LAUREL (baseline and follow-up) and LFRR cohorts (**Table 8**), while being increased compared to matched, clinically unaffected relatives (9-11% Ro/SSA, 1-4% La/SSB) and unaffected HC (2-3% Ro/SSA, 2-3% La/SSB, *p≤0.0117*, **Table 8**). Although clinically unaffected relatives had similar ANA titers and number of SLE-associated autoantibody specificities detected (**Figure 3**), they were more likely than unaffected HC to be positive for autoantibody specificities toward chromatin (10% Rel vs. 0 HC, *p=0.0017*) at baseline (LAUREL), Ro/SSA (11% Rel vs. 3% HC, *p=0.0393*) at follow-up (LAUREL), and Ro/SSA (9% Rel vs. 2% HC, *p=0.0319*) in the LFRR cohort (**Table 8**).

**Table 8:** SLE-Associated Autoantibody Specificities in Lupus Relatives Who Transition to ILE or SLE^a^.

LAUREL Nested Cohort	–>ILE	–>SLE	Lupus Relatives (Rel)	Unaffected HC	ILE/SLE	ILE/SLE/Rel	ILE/SLE/Rel/HC	Rel/HC
Baseline (Prior to SLE Transition)	n=34	n=56	n=154	n=77	*p-value^b^*	*p-value^c^*	*p-value^c^*	*p-value^b^*
**dsDNA**	0	6 (11%)	5 (3%)	6 (8%)	*0.0793*	***0.0275***	*0.0595*	*0.1868*
**Chromatin**	4 (12%)	7 (13%)	16 (10%)	0	*1.0000*	*0.9024*	***0.0226***	***0.0017***
**Ro/SSA**	9 (26%)	14 (25%)	15 (10%)	2 (3%)	*1.0000*	***0.0044***	***<0.0001***	*0.0613*
**La/SSB**	5 (15%)	6 (11%)	6 (4%)	2 (3%)	*0.7415*	***0.0370***	***0.0215***	*0.7220*
**Sm**	0	2 (4%)	0	1 (1%)	*0.5246*	***0.0339***	*0.1073*	*0.3333*
**SmRNP**	1 (3%)	4 (7%)	4 (3%)	2 (3%)	*0.6462*	*0.2937*	*0.4168*	*1.0000*
**RNP**	1 (3%)	8 (14%)	7 (5%)	11 (14%)	*0.1451*	***0.0273***	***0.0164***	***0.0166***
**LAUREL Nested Cohort**	**ILE**	**SLE**	**Lupus Relatives (Rel)**	**Unaffected HC**	**ILE/** **SLE**	**ILE/SLE/** **Rel**	**ILE/SLE/** **Rel/HC**	**Rel/HC**
**Follow-up (After SLE Transition)**	**n=34**	**n=56**	**n=154**	**n=77**	***p-value^b^***	***p-value^c^***	***p-value^c^***	***p-value^b^***
**dsDNA**	1 (3%)	4 (7%)	7 (5%)	6 (8%)	*0.6462*	*0.6305*	*0.6306*	*0.3671*
**Chromatin**	3 (9%)	5 (9%)	2 (1%)	0	*1.0000*	***0.0156***	***0.0028***	*0.5536*
**Ro/SSA**	8 (24%)	15 (27%)	17 (11%)	2 (3%)	*0.8068*	***0.0117***	***0.0001***	***0.0393***
**La/SSB**	5 (15%)	6 (11%)	6 (4%)	2 (3%)	*0.7415*	***0.0370***	***0.0215***	*0.7220*
**Sm**	0	2 (4%)	1 (1%)	1 (1%)	*0.5246*	*0.1846*	*0.3425*	*1.0000*
**SmRNP**	1 (3%)	6 (11%)	4 (3%)	2 (3%)	*0.2469*	***0.0386***	*0.0512*	*1.0000*
**RNP**	2 (6%)	6 (11%)	7 (5%)	11 (14%)	*0.7051*	*0.2575*	*0.0626*	***0.0166***
**LFRR Nested Cohort**	**ILE**	**SLE**	**Lupus Relatives (Rel)**	**Unaffected HC**	**ILE/** **SLE**	**ILE/SLE/** **Rel**	**ILE/SLE/** **Rel/HC**	**Rel/HC**
**Follow-up (After SLE Transition)**	**n=72**	**n=100**	**n=159**	**n=127**	***p-value^b^***	***p-value^c^***	***p-value^c^***	***p-value^b^***
**dsDNA**	1 (1%)	43 (44%)	5 (3%)	3 (3%)	***<0.0001***	***<0.0001***	***<0.0001***	*1.0000*
**Chromatin**	15 (21%)	48 (49%)	10 (6%)	3 (3%)	***0.0002***	***<0.0001***	***<0.0001***	*0.2560*
**Ro/SSA**	19 (26%)	37 (38%)	14 (9%)	2 (2%)	*0.1387*	***<0.0001***	***<0.0001***	***0.0319***
**La/SSB**	11 (15%)	12 (12%)	2 (1%)	2 (2%)	*0.6518*	***0.0001***	***<0.0001***	*0.6504*
**Sm**	4 (6%)	34 (35%)	2 (1%)	0	***<0.0001***	***<0.0001***	***<0.0001***	*0.5194*
**SmRNP**	11 (15%)	42 (43%)	4 (3%)	2 (2%)	***0.0001***	***<0.0001***	***<0.0001***	*1.0000*
**RNP**	11 (15%)	40 (41%)	5 (3%)	1 (1%)	***0.0003***	***<0.0001***	***<0.0001***	*0.4074*

a Seropositivity determined by Bioplex 2200 ANA xMAP assay.

Categorical significance determined by ^b^Chi-square test or ^c^Fisher’s Exact test.p-values in bold are significant at **p≤0.05**.Rel, lupus relatives; HC, healthy controls; ILE, incomplete lupus erythematosus; SLE, systemic lupus erythemtosus.

### 3.5 Alteration of Select Immune Mediators Associated With SLE-CSQ, Serology, and Classification Status in Lupus Relatives

We have previously demonstrated that circulating immune mediator levels are altered prior to the appearance of autoantibody specificities (1, 2) and clinical disease (1, 2, 11) in the development of SLE, and the number and heterogeneous nature of altered immune pathways increases as patients transition to classified SLE (1, 2).

Given the differences in clinical and serologic profiles, as well as participant-reported SLE-CSQ scores in clinically unaffected lupus relatives vs. those who develop ILE or transition to SLE, we assessed which immune mediators were altered relative to these parameters (**Table 9** [lupus relatives only] and **Table S2** [lupus relatives + HC]). We observed most consistent correlation with plasma levels of the pro-inflammatory mediator SCF, soluble TNF superfamily members, particularly the B-lymphocyte activator BLyS, IFN-associated chemokines, and select adaptive mediators, including Th1-type mediators that help drive the production of such chemokines and regulatory mediators IL-10 and active TGF-β. SCF was more likely to be associated with the presence of ACR classification criteria, both prior to (LAUREL baseline) and after SLE classification (LAUREL follow-up and LFRR) whether self-reported (SLE-CSQ score) or medical record confirmed (ACR score), while BLyS was consistently associated with both the presence of ACR classification criteria and the accumulation of autoantibody specificities, both before and after disease classification was reached (**Tables 9** and **S2**). This was also true of IFN-associated chemokines, particularly if healthy individuals were included in the correlation analysis (**Table S2**). The most consistently correlated Th1-type mediator associated with both ACR classification criteria and autoantibody accumulation before and after disease transition was soluble IL-2Rα, while IL-12p70 and IFN-γ had increased correlation with clinical disease after disease transition, particularly in the LFRR cohort (**Tables 9** and **S2**). Curiously, the regulatory mediators IL-10 and active TGF-β presented with a mix of negative correlations to clinical criteria in the LAUREL cohort and positive correlations with both clinical and serologic features in the LFRR cohort (**Tables 9** and **S2**).

**Table 9:** Correlation Between SLE-CSQ Score, ACR Score, or SLE-Associated Autoantibody Specificities and Immune Parameters in Lupus Relatives.

SLE-SCQ Score vs.	LAUREL (BL) Nested Cohort			LAUREL (FU) Nested Cohort			LFRR Nested Cohort		
	Spearman r	95% CI	*p-value^a^*	Spearman r	95% CI	*p-value^a^*	Spearman r	95% CI	*p-value^a^*
**SCF**	0.246	0.121 to 0.364	***0.0001***	0.252	0.127 to 0.369	***<0.0001***	0.160	0.050 to 0.266	***0.0036***
**BLyS**	0.237	0.111 to 0.355	***0.0002***	0.275	0.151 to 0.390	***<0.0001***	0.318	0.214 to 0.414	***<0.0001***
**TNF-α**	-0.051	-0.179 to 0.079	*0.4320*	-0.159	-0.283 to -0.031	***0.0127***	0.121	0.010 to 0.229	***0.0281***
**TNFRI**	0.083	-0.047 to 0.210	*0.1955*	0.154	0.025 to 0.278	***0.0161***	0.162	0.051 to 0.268	***0.0033***
**TNFRII**	0.142	0.012 to 0.266	***0.0271***	0.182	0.054 to 0.304	***0.0045***	0.161	0.051 tp 0.268	***0.0033***
**MCP-1/CCL2**	0.134	0.047 to 0.259	***0.0367***	0.085	-0.045 to 0.212	*0.1856*	0.180	0.070 to 0.285	***0.0010***
**MCP-3/CCL7**	0.182	0.054 to 0.304	***0.0043***	0.043	-0.087 to 0.171	*0.5034*	0.088	-0.023 to 0.197	*0.1108*
**MIG/CXCL9**	0.165	0.037 to 0.289	***0.0096***	0.008	-0.121 to 0.138	*0.5034*	0.048	-0.063 to 0.159	*0.3830*
**IP-10/CXCL10**	0.049	-0.081 to 0.177	*0.4452*	-0.071	-0.198 to 0.059	*0.2724*	0.158	0.048 to 0.265	***0.0039***
**IL-2Rα**	0.148	0.019 to 0.272	***0.0210***	0.189	0.061 to 0.310	***0.0031***	0.225	0.117 to 0.328	***<0.0001***
**IL-12p70**	-0.021	-0.150 to 0.108	*0.7416*	-0.119	-0.244 to 0.011	*0.0641*	0.186	0.077 to 0.291	***0.0007***
**IFN-γ**	-0.035	-0.163 to 0.095	*0.5902*	-0.087	-0.214 to 0.043	*0.1767*	0.164	0.054 to 0.270	***0.0028***
**IL-10**	-0.078	-0.205 to 0.052	*0.2265*	-0.148	-0.272 to -0.019	***0.0206***	0.201	0.092 to 0.305	***0.0002***
**Active TGF-β**	-0.138	-0.262 to -0.009	***0.0314***	-0.127	-0.252 to 0.002	***0.0474***	0.062	-0.050 to 0.172	*0.2648*
**ACR Score vs.**	**Spearman r**	**95% CI**	***p-value^a^***	**Spearman r**	**95% CI**	***p-value^a^***	**Spearman r**	**95% CI**	***p-value^a^***
**SCF**	0.298	0.176 to 0.412	***<0.0001***	0.271	0.147 to 0.387	***<0.0001***	0.081	-0.030 to 0.190	*0.1411*
**BLyS**	0.214	0.087 to 0.334	***0.0008***	0.264	0.139 to 0.380	***<0.0001***	0.398	0.300 to 0.487	***<0.0001***
**TNF-α**	0.013	-0.117 to 0.142	*0.8426*	-0.177	-0.299 to -0.049	***0.0057***	-0.008	-0.119 to 0.103	*0.8809*
**TNFRI**	0.017	-0.113 to 0.146	*0.7911*	0.093	-0.037 to 0.219	*0.1497*	0.227	0.117 to 0.329	***<0.0001***
**TNFRII**	0.062	-0.068 to 0.189	*0.3385*	0.103	-0.026 to 0.230	*0.1071*	0.205	0.097 to 0.309	***0.0002***
**MCP-1/CCL2**	0.129	0.000 to 0.254	***0.0439***	0.183	0.055 to 0.305	***0.0041***	0.059	-0.052 to 0.169	*0.2806*
**MCP-3/CCL7**	0.187	0.059 to 0.309	***0.0034***	0.101	-0.029 to 0.227	*0.1157*	-0.085	-0.194 to 0.027	*0.1243*
**MIG/CXCL9**	0.063	-0.067 to 0.191	*0.3258*	0.032	-0.097 to 0.161	*0.6155*	0.078	-0.034 to 0.187	*0.1584*
**IP-10/CXCL10**	-0.045	-0.173 to 0.085	*0.4838*	-0.061	-0.189 to 0.069	*0.3412*	0.216	0.107 to 0.319	***<0.0001***
**IL-2Rα**	0.119	-0.011 to 0.244	*0.0642*	0.212	0.085 to 0.332	***0.0009***	0.288	0.183 to 0.386	***<0.0001***
**IL-12p70**	0.020	-0.109 to 0.149	*0.7526*	-0.180	-0.303 to -0.052	***0.0047***	0.198	0.089 to 0.302	***0.0003***
**IFN-γ**	0.001	-0.129 to 0.130	*0.9928*	-0.175	-0.298 to -0.047	***0.0061***	0.048	-0.063 to 0.158	*0.3833*
**IL-10**	-0.064	-0.192 to 0.065	*0.3163*	-0.219	-0.339 to -0.093	***0.0006***	0.252	0.145 to 0.353	***<0.0001***
**Active TGF-β**	-0.113	-0.239 to 0.017	*0.0788*	-0.192	-0.314 to -0.065	***0.0026***	0.021	-0.090 to 0.132	*0.7017*
**# SLE-associated AutoAbs vs.**	**Spearman r**	**95% CI**	***p-value^a^***	**Spearman r**	**95% CI**	***p-value^a^***	**Spearman r**	**95% CI**	***p-value^a^***
**SCF**	0.136	0.007 to 0.261	***0.0339***	0.789	-0.051 to 0.206	*0.2194*	0.068	-0.043 to 0.178	*0.2182*
**BLyS**	0.326	0.205 to 0.437	***<0.0001***	0.199	0.071 to 0.320	***0.0018***	0.328	0.225 to 0.424	***<0.0001***
**TNF-α**	0.036	-0.094 to 0.164	*0.5799*	0.010	-0.119 to 0.139	*0.8749*	0.124	0.013 to 0.231	***0.0246***
**TNFRI**	0.063	-0.067 to 0.190	*0.3296*	0.570	-0.073 to 0.185	*0.3750*	0.182	0.072 tp 0.287	***0.0009***
**TNFRII**	0.155	0.026 to 0.279	***0.0153***	0.083	-0.047 to 0.210	*0.1961*	0.230	0.122 to 0.333	***<0.0001***
**MCP-1/CCL2**	0.194	0.066 to 0.315	***0.0024***	0.899	-0.040 to 0.217	*0.1617*	0.086	-0.026 to 0.195	*0.1198*
**MCP-3/CCL7**	0.260	0.136 to 0.377	***<0.0001***	0.104	-0.028 to 0.288	*0.1141*	-0.024	-0.133 tp 0.089	*0.6842*
**MIG/CXCL9**	0.207	0.079 to 0.327	***0.0012***	0.200	0.073 to 0.321	***0.0017***	0.255	0.148 to 0.356	***<0.0001***
**IP-10/CXCL10**	0.222	0.095 to 0.341	***0.0005***	0.138	0.008 to 0.262	***0.0318***	0.366	0.265 to 0.458	***<0.0001***
**IL-2Rα**	0.238	0.112 to 0.356	***0.0002***	0.244	0.119 to 0.362	***0.0001***	0.192	0.083 to 0.297	***0.0004***
**IL-12p70**	-0.005	-0.134 to 0.125	*0.9405*	0.090	-0.216 to 0.040	*0.1636*	0.252	0.145 to 0.353	***<0.0001***
**IFN-γ**	0.032	-0.098 to 0.160	*0.6230*	-0.059	-0.187 to 0.071	*0.3586*	0.132	0.021 to 0.239	***0.0166***
**IL-10**	0.017	-0.112 to 0.146	*0.7864*	-0.078	-0.205 to 0.052	*0.2269*	0.285	0.180 to 0.384	***<0.0001***
**Active TGF-β**	0.053	-0.077 to 0.181	*0.4086*	-0.091	-0.218 to 0.038	*0.1551*	0.164	0.053 to 0.270	***0.0029***

a Spearman correlation Bonferroni corrected **p≤0.0036.**p-values in bold are significant at **p≤0.05**.BL, baseline; BLyS, B lymphocyte stimulator; FU, follow-up; HC, healthy controls; MCP-1, monocyte chemoattractant protein -1; MIG, monokine induced by gamma interferon; IP-10, interferon-γ-inducible protein-10; SCF, stem cell factor; TNF, tumor necrosis factor; TNFR, tumor necrosis factor receptor; TGF-β, transforming growth factor-β ; LAUREL, Lupus Autoimmunity in Relatives; LFRR, Lupus Family Registry and Repository.

**Figure 5:**
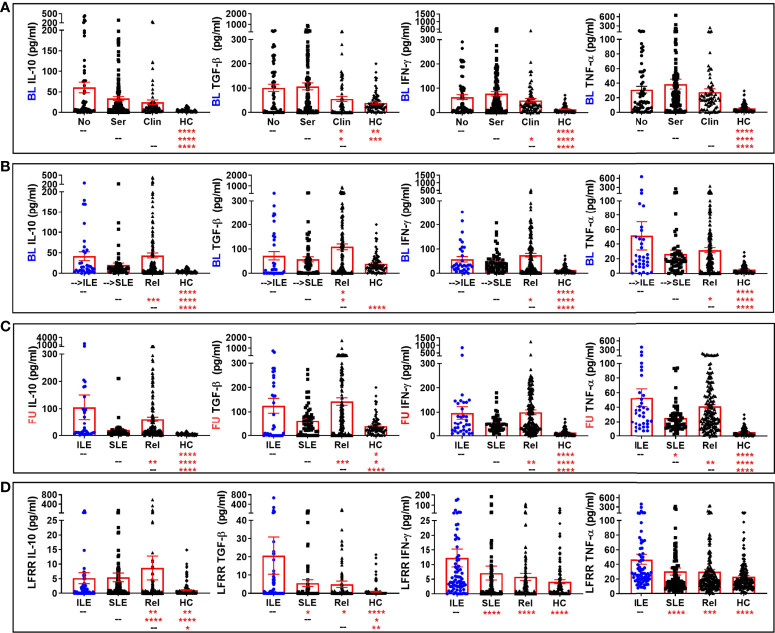
Altered regulatory and select Th1-type mediators in lupus relatives who develop ILE or transition to SLE. Lupus relatives and matched, unaffected healthy controls (HC) were evaluated for plasma levels of evaluated for plasma levels of IL-10 (*1^st^ column*), active TGF-β (*2^nd^ column*), IFN-γ (*3^rd^ column*), and soluble TNF-α (*4^th^ column*) in **(A)** LAUREL cohort at baseline meeting No ACR criteria (No), only serologic ACR criteria (Ser), or clinical ACR criteria (Clin) vs. matched, unaffected HC and **(B–D)** lupus relatives who developed ILE (ILE), transitioned to SLE (SLE), or remained clinically unaffected (Rel) vs. matched healthy controls (HC) in **(B)** LAUREL cohort at baseline (pre-transition), **(C)** LAUREL cohort at follow-up (post-transition), and **(D)** LFRR confirmatory cohort (post-transition). Mean ± SEM. ****p<0.0001; ***p<0.001; **p<0.01; *p<0.05 by Kruskal-Wallis with Dunn’s multiple comparison.

We compared levels of these apparently altered immune mediators prior to (LAUREL baseline) and after disease transition (LAUREL FU and LFRR) in lupus relatives who remained clinically unaffected, developed clinical symptoms that either resulted in ILE or SLE classification, as well as matched healthy individuals (**Figures 4**, **5** and **S5**). Prior to disease transition, levels of pro-inflammatory mediators SCF, BLyS, MCP-3, and IL-2Rα (**Figure 4A**), as well as MCP-1 and MIG (**Figure S5A**) were highest in those lupus relatives in the LAUREL cohort who met clinical ACR criteria at baseline (*p<0.05*). With the exception of MCP-1, these mediators remained elevated pre- and post-transition in lupus relatives who developed ILE or SLE in both the LAUREL (**Figures 4B, C** **)** and LFRR (**Figure 4D**) cohorts. Of note, IFN-associated chemokines MCP-1 and IP-10, as well as Th1-type mediator IL-12p70, were increased in lupus relatives irrespective of disease transition status, while MIG was more likely to be increased in lupus relatives who developed ILE. TNFRII was increased in all lupus relatives, while TNFRI was equally increased in relatives developing ILE or SLE in the LAUREL cohort, with both further differentiating relatives who entered the LFRR with classified SLE (**Figure S5**).

Conversely, the regulatory mediators IL-10 and active TGF-β, as well as IFN-γ, were lowest in HC and lupus relatives in the LAUREL cohort who met clinical ACR criteria at baseline (**Figure 5A**). These mediators, as well as TNF-α, were highest in the LAUREL cohort at baseline and follow-up in those lupus relatives who remained clinically unaffected or only developed ILE and did not transition to classified SLE (**Figures 5B, C** **)**. In the LFRR cohort, IL-10 was highest in lupus relatives who were clinically unaffected, while active TGF-β, as well as IFN-γ and TNF-α, were elevated in lupus relatives with ILE (**Figure 5D**). These data suggest that some pro-inflammatory mediators are able to possibly overwhelm immune regulation to drive the development and pathogenesis of SLE, while others may be offset by regulatory mediators to either prevent clinical disease or stall it from transitioning to classified SLE.

**Figure 6:**
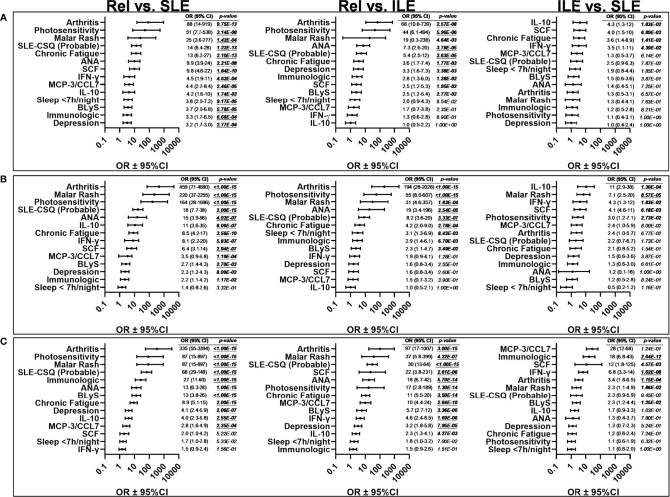
Effect size of informative variables that distinguish lupus relatives prior to and after disease transition in the LAUREL and LFRR cohorts. Odds ratios (± 95% CI) were determined by Fisher exact test for lupus relatives (Rel) vs. relatives who transitioned to SLE, prior to SLE classification in the LAUREL baseline cohort **(A**, Rel vs. SLE**)**, for clinical, serologic, and immunologic differentiating variables as outlined in **Table S3**. Bonferroni’s correction for multiple comparison was applied to all significant variables (*p<0.05*); the 14 variables with *p≤0.0036* were considered significant for differentiating Rel vs. SLE **(A)** prior to disease transition. These same variables were assessed for effect size and significance comparing Rel vs ILE and ILE vs SLE prior to disease transition in the LAUREL cohort at baseline **(A)**, as well as Rel vs SLE, Rel vs ILE, and ILE vs SLE after disease transition in the LAUREL cohort at follow-up **(B)**, as well as the LFRR confirmatory cohort **(C)**.

To determine how well soluble mediators differentiated unaffected relatives vs. those who developed ILE or transitioned to SLE, we determined positive/negative cut-off values between Rel and SLE in each cohort based on the Youden Index that maximizes sensitivity and specificity (55). We then compared size effects (odds ratios) across 14 parameters across type 2 symptoms, ACR criteria, SLE-CSQ scores, and soluble mediators that remained significant after Bonferroni correction (*p≤0.0036*) when comparing unaffected relatives vs. relatives in the LAUREL cohort at baseline who would transition to SLE (**Figure 6A**, left panel). SCF, IFN-γ, IL-10, and BLyS, alongside reported type 2 symptoms chronic fatigue, depression, and sleep disturbances, probable SLE (SLE-CSQ score ≥4) based on the SLE-CSQ questionnaire, as well as ACR criteria arthritis, photosensitivity, immunologic criteria, and ANA positivity differentiated unaffected Rel vs. relatives who would transition to SLE prior to disease classification. Eleven out of 14 parameters remained significant post-SLE classification in both the LAUREL cohort at follow-up (**Figure 6B**, left panel) and the confirmatory LFRR cohort (**Figure 6C**, left panel). Clinical ACR criteria, positive ANA, and a probable SLE-CSQ score, alongside SCF and BLyS, consistently differentiated unaffected relatives vs. those who developed ILE (**Figures 6A–C**, middle panel), while IL-10, SCF, and ACR criteria best differentiated ILE vs. SLE across the cohorts (**Figures 6A–C**, right panel).

## 4 Discussion

Reliably identifying those at highest risk of developing lupus clinical features and/or transitioning to classified SLE for early intervention vs. those who do not advance beyond latent autoimmunity remains challenging. Despite the presence of familial genetics (61) and more than two-fold increased frequency of antinuclear antibody (ANA) positivity (51) compared to the general population (62), a considerable majority of lupus relatives will never transition to classified SLE (63, 64). Many will remain clinically unaffected in a state of persistent latent autoimmunity that does not progress beyond serologic features (65, 66). Others may also develop clinical features of SLE with heightened risk of permanent organ damage (67), yet never reach disease classification (41). In both unique cohorts evaluated in the current study (11, 46, 68), lupus relatives without classified disease were more likely to be parents, children, or siblings of SLE patients, while those who had transitioned to classified SLE were noted to be more distant relatives. Although somewhat surprising, other studies have noted similar findings, with adult-onset SLE among families increased among non-first degree relatives (9, 10, 60).

Also of note was that lupus relatives who transitioned to SLE in the LAUREL cohort were older than those with classified disease in the confirmatory LFRR nested cohort, possibly because they were recruited into the LAUREL cohort prior to disease transition at baseline and were more likely to be of European American descent (11, 68). Similar to other studies, we noted in the current study that the potentially later-onset SLE in the LAUREL cohort included more males (69) and more European Americans (70), with a somewhat milder presentation vs. SLE patients evaluated from the LFRR confirmatory cohort, including less renal, hematologic, and immunologic criteria and more mucocutaneous criteria post-transition in the LAUREL cohort (70–73). Yet, those with classified SLE in the LAUREL and LFRR cohorts met roughly the same number of ACR criteria, and others have shown that damage accrual is similar between early- and late-onset SLE (69, 72), with the potential for more co-morbidities in late-onset SLE (70). These findings reinforce the need for astute long-term follow-up of lupus relatives at highest risk of disease transition.

For many, transition to classified SLE has an insidious clinical onset that can be difficult to pinpoint (70), especially since some of the first patient-reported symptoms may include non-specific “type 2” (33, 35) SLE-associated symptoms such as fatigue, anxiety, depression, chronic headaches, and sleep disturbances (36, 37, 74, 75). Although these were more likely to be present in pre- and post-classification lupus relatives who also met clinical ACR criteria in the LAUREL and LFRR cohorts, with fatigue more prevalent in pre-SLE relatives at baseline, they were also more frequent at baseline and follow-up in clinically unaffected relatives compared to HC in the LAUREL cohort. These findings reinforce the notion of intertwining of type 2 and type 1 (inflammatory/clinical) features in SLE (33, 35), and justify the need for more SLE-specific symptom screening in lupus relatives. Of note, SLE-CSQ scores were consistently increased in lupus relatives and HC in both the LAUREL and LFRR cohorts who reported type 2 symptoms, with the highest scores in relatives who also presented with clinical ACR criteria at baseline and developed ILE or transitioned to SLE at follow-up in LAUREL and the LFRR. Yet, SLE-CSQ scores were also increased in clinically unaffected relatives compared to matched HC.

SLE-CSQ scores were highly correlative with number of medical record confirmed ACR criteria met in both cohorts, even before SLE transition, supporting the utility of SLE-CSQ as a clinical screening tool (11, 41). The increase in SLE-CSQ scores associated with type 2 symptoms suggests that there may also be additional underlying alternate or concurrent non-SLE processes. One candidate that may be present in both clinically unaffected relatives and those who develop ILE or SLE is fibromyalgia, which has been previously noted in SLE patients with either active or inactive disease who experience type 2 symptoms (33). Many fibromyalgia patients are also ANA positive, yet previous studies suggest that ANA positivity is not necessarily predictive of SLE or other autoimmune disease development (76, 77), similar to what we have observed in lupus relatives [(11) and current study]. Another candidate, with or without fibromyalgia, is undifferentiated connective tissue disease (UCTD) (78), particularly in unaffected lupus relatives. Unlike their ILE counterparts, who met both serologic and clinical classification criteria for SLE, and a number of whom were being treated with immunosuppressive medication, clinically unaffected lupus relatives exhibited only ANA positivity and immunologic/serologic manifestations, usually anti-cardiolipin autoantibody positivity. That both ILE and clinically unaffected lupus relatives exhibited increased levels of regulatory immune mediators suggests that the presence of clinical classification criteria may differentiate ILE from UCTD (37, 78) and is supported by the presence of arthritis or photosensitivity being among the greatest differentiators of lupus relatives who remained clinically unaffected or developed ILE, whether in the LAUREL cohort at baseline or follow-up or in the confirmatory LFRR cohort.

Although differences in ANA titer or autoantibody specificity accumulation were not noted with the presence of type 2 symptoms (data not shown), except for sleep disturbances, where no patterns of immune mediator changes were found, there was a consistent increase in plasma BLyS levels, particularly among lupus relatives reporting type 2 symptoms who remained clinically unaffected or only developed ILE. Conversely, increased plasma levels of IL-10 were found in lupus relatives who did *not* report type 2 symptoms, particularly for fatigue. These findings suggest a unique opportunity for intervention in lupus relatives reporting type 2 symptoms with elevated BLyS and/or decreased IL-10 levels, as belimumab has been shown to improve fatigue and quality of life measures in SLE patients (79, 80), while non-pharmacologic modalities such as physical (81, 82) and mindfulness (83) exercises have been shown to increase anti-inflammatory IL-10 levels and decrease fatigue and other type 2 symptoms. Although no immune mediators were found to be associated with sleep disturbances, we observed in the current study that sleep disturbances were more prevalent in lupus relatives meeting clinical ACR criteria at baseline (pre-transition) and that those averaging less than seven hours of sleep/night were more likely to transition to SLE [(57, 84) and current study].

Given that lupus relatives who remain clinically unaffected with respect to SLE classification may have other underlying symptoms that would benefit from clinical assessment and intervention, and that individuals with ILE, even if they never reach SLE classification, are at risk for accumulating organ damage (69, 72), screening approaches to identify lupus relatives for early intervention trials and longitudinal assessment studies would be beneficial to both more closely dissect and address immune dysregulation prior to disease classification (85) and potentially reduce the socioeconomic burden of SLE (86). ANA positivity alone, whether in familial (9–11, 66) or non-familial (1, 87) cohorts, is not predictive of who will develop ILE or transition to SLE. Additionally utilizing the SLE-CSQ, that was found to be strongly associated with medical record confirmed cumulative ACR scores, would add specificity for SLE and negative predictive value without substantial increase in administrative burden, particularly if screening for lupus relatives with SLE-CSQ scores of 3 (possible lupus) or more (probable lupus) (68).

In addition, screening for immune pathway dysregulation in conjunction with ANA positivity may improve our ability to identify individuals at high risk for developing clinical disease (1, 11, 41). In a more limited subset of lupus relatives in the LAUREL cohort, we have previously shown that the pro-inflammatory mediator SCF was an independent predictor of transition to classified SLE (41), with confirmation of enhanced SCF levels in relatives who developed ILE or transitioned to SLE in the expanded group of relatives in the LAUREL and LFRR cohort in the current study. SCF interacts with the receptor, c-kit, to enhance pro-inflammatory adaptive immunity (32, 88) that drives downstream effector mediators that include MCP chemokines, MCP-1 and MCP-3 (31), that were increased in lupus relatives, including those with clinical disease. In addition to being associated with reported type 2 symptoms, plasma levels of BLyS were also observed to be elevated in lupus relatives, particularly those meeting clinical disease criteria who developed ILE or transitioned to SLE. BLyS is produced in response to both type I IFN (IFN-α) (89), a heritable risk factor in SLE (13), and type II IFN (IFN-γ) (21), a Th1-type cytokine affected by signaling through IL-2Rα (90, 91), the soluble form of which was similarly increased in the current study. In addition to its association with SLE pathogenesis (22) and disease activity and flare (92, 93), BLyS has been shown in previous studies to be elevated as patients transition from autoantibody positivity to clinical disease and transition to classified SLE (1, 2), with blockade of BLyS (23, 24), as well as type I IFN receptors (25, 26) and IFN-γ (27) that drive BLyS, having the potential to improve disease outcomes in subsets of SLE patients.

In contrast, the regulatory mediator IL-10, observed to be decreased in lupus relatives with type 2 symptoms, along with active TGF-β, previously shown to be a negative predictor of SLE transition in a more limited subset of lupus relatives in the LAUREL cohort (41), were both increased in lupus relatives *without* clinical ACR criteria at baseline (LAUREL), as well as clinically unaffected relatives and relatives who only developed ILE, but did not have classified SLE at follow-up (LAUREL and LFRR). That lupus relatives who only developed ILE also had elevated levels of regulatory mediators may explain the mix of negative and positive correlations to SLE-CSQ scores, ACR scores, and autoantibody specificity accumulation in the LAUREL and LFRR cohorts in the current study. Curiously, we observed similar increased levels of TNF-α and IFN-γ in clinically unaffected relatives and relatives with ILE, but not classified SLE, in the current study. One possible explanation is that relatives with classified SLE were more likely to be on immune modifying treatments that may decrease these mediators, particularly if these patients were well managed. We have previously shown that both TNF-α and IFN-γ are maintained at lower levels in the periphery during periods of non-flare, with rising levels precipitating imminent clinical disease flare (18, 19). For clinically unaffected relatives and those who developed ILE, the Th1-type adaptive mediator IFN-γ is among the earliest dysregulated mediators detected in pre-clinical SLE (1, 2), with TNF-α belonging to the same Th1-type cytokine group. The concurrent upregulation of regulatory mediators in these same lupus relatives has the potential to offset underlying basal inflammation in these individuals, while a likely feed-forward effect of accumulating altered inflammatory pathways takes place in those who transition to classified SLE (1, 2).

There are a number of limitations in the current study. Due to the vast majority of lupus relatives entering both the LAUREL and confirmatory LFRR cohorts years before either the SLICC (52) or EULAR/ACR (94) SLE classification criteria were published, it was necessary to utilize the 1997 ACR classification criteria (47, 48) in the current study. Yet, there were similarities in both ACR scores and the recently published SLERPI (50) scores across both LAUREL and the confirmatory LFRR cohorts. The use of unique cohorts necessitated utilization of the nested LFRR cohort as a confirmatory cohort for the follow-up findings in LAUREL. The difference in timing of biological assessments between the cohorts, particularly soluble immune mediators requiring research-use-only multiplex immunoassay platforms that are highly sensitive and specific while sample sparing, but known for inter-user and inter-lot variability (95), precluded the combining of datasets for analysis. Despite this caveat, immune dysregulation noted in LAUREL was largely recapitulated in the confirmatory LFRR cohort. Despite being able to tease out type 2 symptoms in both cohorts, other self-reported data, such as smoking (96) and alcohol consumption (97), were not widely available for analysis in the current study. That being said, a previous study assessing a subset of SLE patients, lupus relatives, and healthy controls with available self-reported smoking data in the LFRR found no association with increased autoantibody production (98). Finally, the LAUREL cohort only provided a single follow-up time point, and unlike the Department of Defense SLE cohort (1, 2), was not able to provide serially collected longitudinal samples for assessment as lupus relatives transition to classified SLE.

Identifying lupus relatives at risk of transitioning to SLE vs. those who may remain in a state of latent autoimmunity is necessary to decrease the rate of early organ damage for those who transition (5) while reducing the necessity for multiple and/or immunosuppressant treatments that perpetuate morbidity and increased healthcare costs (86). In addition to self-reported symptoms as well as serologic and clinical classification criteria, we found in the current study that immune mediator alterations also differentiate lupus relatives who develop ILE or SLE compared to clinically unaffected relatives and HC. Early intervention in SLE may be most effective before the immune system enters a feed-forward, self-sustaining cycle of broken tolerance that results in clinical disease and transition to classified SLE (99). In addition to its potential for treating lupus relatives with type 2 symptoms, discussed above, increased levels of BLyS associated with classification status and the success of belimumab in subsets of SLE patients with classified disease (23) makes this drug a potential steroid-sparing candidate for early intervention in lupus relatives at increased risk of developing clinical disease, particularly those without pre-existing organ damage (100). For those lupus relatives with ILE who meet some clinical ACR criteria, but have not reached SLE classification, hydroxychloroquine may be a viable early intervention candidate (101), with evidence of delayed transition to classified SLE (7) and clinical improvement in patients with ILE (8). Adequate screening using a combination of self-reported assessments and serological immune components, coupled with longitudinal monitoring and early intervention strategies may be the key to maintain clinically unaffected lupus relatives and delaying or preventing disease transition in relatives who already meet clinical classification criteria.

## Data Availability Statement

The raw data supporting the conclusions of this article will be made available by the authors, without undue reservation.

## Ethics Statement

The studies involving human participants were reviewed and approved by Institutional Review Board, OMRF and MUSC. The patients/participants provided their written informed consent to participate in this study.

## Author Contributions

MEM designed and carried out experiments, completed data analysis, and principally wrote manuscript. KAY, JMG, and JMN provided experimental and editorial guidance. DLK, GSG, MHW, MLI, DJW, DRK, JBH, and JAJ provided patient data and samples for the LAUREL and LFRR cohorts, as well as editorial guidance. JAJ provided additional support in addition to experimental and editorial guidance. All authors contributed to the article and approved the submitted version.

## Funding

This study was supported by the National Institute of Allergy, Immunology and Infectious Diseases, Office of Research on Women’s Health, National Institute of General Medical Sciences, and the National Institute of Arthritis, Musculoskeletal and Skin Diseases under award numbers U01AI101934, U19AI082714, UM1AI144292, P30AR073750, U54GM104938, and P30GM103510. This material is also the result of work supported with resources and the use of facilities through the Department of Veterans Affairs. This publication is the sole responsibility of the authors and does not represent the views of the National Institutes of Health or the Department of Veterans Affairs. This work was also supported by the OMRF Lou C. Kerr Chair in Biomedical Research to JAJ.

## Author Disclaimer

The views expressed in this article are those of the authors and do not necessarily reflect the official policy or position of the Department of the Navy, Department of the Army, US Armed Forces Department of Defense, or the US Government.

## Conflict of Interest

The authors declare that the research was conducted in the absence of any commercial or financial relationships that could be construed as a potential conflict of interest.

## Publisher’s Note

All claims expressed in this article are solely those of the authors and do not necessarily represent those of their affiliated organizations, or those of the publisher, the editors and the reviewers. Any product that may be evaluated in this article, or claim that may be made by its manufacturer, is not guaranteed or endorsed by the publisher.
