# Supplementary material for: Pre-Clinical Autoimmunity in Lupus Relatives: Self-Reported Questionnaires and Immune Dysregulation Distinguish Relatives Who Develop Incomplete or Classified Lupus From Clinically Unaffected Relatives and Unaffected, Unrelated Individuals
Source: Front Immunol. 2022 Jun 3;13:866181. doi: 10.3389/fimmu.2022.866181 (PMC9203691; doi:10.3389/fimmu.2022.866181)
Supplement: Supplementary file 1 [file DataSheet_1.pdf]

frontiers

Supplementary Material

Table S1. Plasma Soluble Mediators Assessed by xMAP Assay in Study

**Innate**	**Th1-like**	**NGF/TNFR Superfamily**
IL-1α	IL-12 (p70)	BLyS*
IL-1β	IFN-γ	APRIL*
IL-1RA	IL-2	sCD40L
IFN-α	IL-2Rα	sFas
IFN-β		sFasL
G-CSF	**Th-17 like**	TNF-α
	IL-6	TNFRI (p55)
**Homeostasis**	IL-23	TNFRII (p75)
IL-7	IL-17A	TRAIL
IL-15	IL-21	NGFβ
**Other**	**Th2-like**	**Chemokine/Adhesion molecules**
LIF	IL-4	IL-8/CXCL8
PAI-1	IL-5	IP-10/CXCL10
PDGF-BB	IL-13	RANTES/CCL5
Resistin		MIP-1α/CCL3
Leptin	**Regulatory**	MIP-1β/CCL4
SCF	IL-10	MCP-1/CCL2
	TGF-β	MCP-3/CCL7
		GROα/CXCL1
		SDF-1/CXCL12
		MIG/CXCL9
		Eotaxin/CCL11
		ICAM-1
*assessed by ELISA		VCAM-1
		sE-selectin
		VEGF-A

Table S2. Correlation Between SLE-CSQ Score, ACR Score, or SLE-Associated Autoantibody Specificities and Immune Parameters in Lupus Relatives and Matched HC

	LAUREL (BL) Nested Cohort			LAUREL (FU) Nested Cohort			LFRR Nested Cohort		
SLE-SCQ Score vs.	Spearman r	95% confidence interval	*p-value*[a]	Spearman r	95% confidence interval	*p-value*[a]	Spearman r	95% confidence interval	*p-value*[a]
ACR Score	0.555	0.472 to 0.629	≤0.0001	0.594	0.516 to 0.662	≤0.0001	0.705	0.654 to 0.750	≤0.0001
ANA titer	0.389	0.289 to 0.480	≤0.0001	0.357	0.254 to 0.451	≤0.0001	0.499	0.425 to 0.567	≤0.0001
# of SLE-associated AutoAbs	0.158	0.046 to 0.266	0.0046	0.098	-0.014 to 0.209	0.0782	0.397	0.314 to 0.474	≤0.0001
SCF	0.084	-0.029 to 0.195	0.1327	0.088	-0.025 to 0.199	0.1140	0.231	0.140 to 0.319	≤0.0001
BLyS	0.365	0.264 to 0.459	≤0.0001	0.393	0.293 to 0.484	≤0.0001	0.332	0.246 to 0.414	≤0.0001
TNF-α	0.199	0.087 to 0.304	0.0003	0.230	0.121 to 0.334	≤0.0001	0.166	0.073 to 0.257	0.0004
TNFRI	0.141	0.029 to 0.250	0.0113	0.055	-0.028 to 0.167	0.3235	0.205	0.113 to 0.293	≤0.0001
TNFRII	0.079	-0.034 to 0.190	0.1599	0.029	-0.084 to 0.141	0.6034	0.217	0.125 to 0.305	≤0.0001
MCP-1/CCL2	0.329	0.224 to 0.426	≤0.0001	0.216	0.106 to 0.321	≤0.0001	0.208	0.116 to 0.297	≤0.0001
MCP-3/CCL7	0.174	0.062 to 0.281	0.0018	0.104	-0.008 to 0.215	0.0521	0.167	0.074 to 0.257	0.0003
MIG/CXCL9	0.290	0.183 to 0.390	≤0.0001	0.153	0.041 to 0.261	0.0059	0.157	0.063 to 0.247	0.0008
IP-10/CXCL10	0.241	0.132 to 0.345	≤0.0001	0.129	0.016 to 0.238	0.0210	0.228	0.136 to 0.315	≤0.0001
IL-2Rα	0.124	0.012 to 0.234	0.0262	0.085	-0.028 to 0.196	0.1300	0.256	0.165 to 0.342	≤0.0001
IL-12p70	0.081	-0.032 to 0.192	0.1455	0.146	0.034 to 0.254	0.0090	0.241	0.150 to 0.328	≤0.0001
IFN-γ	0.179	0.067 to 0.286	0.0013	0.253	0.144 to 0.355	≤0.0001	0.176	0.083 to 0.266	0.0002
IL-10	0.150	0.038 to 0.258	0.0072	0.198	0.087 to 0.304	0.0004	0.223	0.131 to 0.310	≤0.0001
Active TGF-β	-0.070	-0.182 to 0.0426	0.2083	0.019	-0.094 to 0.131	0.7390	0.123	0.029 to 0.215	0.0083
ACR Score vs.	Spearman r	95% confidence interval	*p-value*[a]	Spearman r	95% confidence interval	*p-value*[a]	Spearman r	95% confidence interval	*p-value*[a]
ANA titer	0.698	0.636 to 0.752	≤0.0001	0.576	0.495 to 0.647	≤0.0001	0.747	0.702 to 0.786	≤0.0001
# of SLE-associated AutoAbs	0.186	0.074 to 0.292	0.0008	0.210	0.010 to 0.315	≤0.0001	0.531	0.459 to 0.595	≤0.0001
SCF	0.159	0.047 to 0.267	0.0043	0.112	-0.001 to 0.222	0.0454	0.185	0.092 to 0.275	≤0.0001
BLyS	0.327	0.222 to 0.424	<0.0041	0.397	0.297 to 0.487	≤0.0001	0.357	0.272 to 0.437	≤0.0001
TNF-α	0.207	0.096 to 0.312	0.0002	0.230	0.120 to 0.334	≤0.0001	0.057	-0.037 to 0.151	0.2217
TNFRI	0.051	-0.061 to 0.163	0.3606	-0.022	-0.134 to 0.091	0.6939	0.234	0.143 to 0.321	≤0.0001
TNFRII	0.030	-0.083 to 0.142	0.5893	-0.034	-0.146 to 0.079	0.5424	0.229	0.138 to 0.317	≤0.0001
MCP-1/CCL2	0.255	0.146 to 0.357	≤0.0001	0.267	0.159 to 0.369	≤0.0001	0.132	0.038 to 0.223	0.0048
MCP-3/CCL7	0.183	0.071 to 0.289	0.0010	0.144	0.032 to 0.252	0.0099	0.051	-0.043 to 0.145	0.2769
MIG/CXCL9	0.192	0.050 to 0.269	0.0037	0.155	0.043 to 0.263	0.0053	0.175	0.081 to 0.264	0.0002
IP-10/CXCL10	0.116	0.003 to 0.226	0.0378	0.108	-0.004 to 0.218	0.0533	0.273	0.184 to 0.358	≤0.0001
IL-2Rα	0.092	-0.020 to 0.203	0.0983	0.083	-0.030 to 0.193	0.1400	0.269	0.179 to 0.354	≤0.0001
IL-12p70	0.128	0.015 to 0.237	0.0219	0.120	0.008 to 0.230	0.0311	0.224	0.133 to 0.312	≤0.0001
IFN-γ	0.199	0.088 to 0.305	0.0003	0.216	0.106 to 0.321	≤0.0001	0.104	0.010 to 0.196	0.0264
IL-10	0.131	0.019 to 0.240	0.0186	0.158	0.046 to 0.266	0.0046	0.251	0.160 to 0.337	≤0.0001
Active TGF-β	-0.035	-0.147 to 0.078	0.5285	-0.035	-0.135 to 0.090	0.6897	0.090	-0.005 to 0.183	0.0548
# SLE-associated AutoAbs vs.	Spearman r	95% confidence interval	*p-value*[a]	Spearman r	95% confidence interval	*p-value*[a]	Spearman r	95% confidence interval	*p-value*[a]
ANA titer	0.256	0.158 to 0.367	≤0.0001	0.283	0.176 to 0.383	≤0.0001	0.551	0.482 to 0.613	≤0.0001
SCF	0.101	-0.012 to 0.211	0.0715	0.049	-0.063 to 0.161	0.3759	0.143	0.050 tp 0.235	0.0021
BLyS	0.270	0.163 to 0.372	≤0.0001	0.168	0.057 to 0.276	0.0025	0.311	0.223 to 0.394	≤0.0001
TNF-α	0.053	-0.060 to 0.165	0.3414	0.021	-0.092 to 0.134	0.7048	0.154	0.060 to 0.244	0.0010
TNFRI	0.060	-0.054 to 0.171	0.2881	0.046	-0.067to 0.158	0.4114	0.212	0.120 to 0.301	≤0.0001
TNFRII	0.139	0.027 to 0.248	0.0126	0.083	-0.029 to 0.195	0.1335	0.247	0.157 to 0.334	≤0.0001
MCP-1/CCL2	0.147	0.035 to 0.256	0.0083	0.046	-0.067 to 0.158	0.4070	0.151	0.057 to 0.242	0.0012
MCP-3/CCL7	0.177	0.066 to 0.284	0.0014	0.046	-0.067 to 0.158	0.4152	0.075	-0.019 to 0.168	0.1082
MIG/CXCL9	0.168	0.056 to 0.275	0.0026	0.156	0.044 to 0.264	0.0051	0.285	0.196 to 0.369	≤0.0001
IP-10/CXCL10	0.168	0.056 to 0.275	0.0026	0.090	-0.023 to 0.200	0.1090	0.370	0.285 to 0.449	≤0.0001
IL-2Rα	0.237	0.128 to 0.341	≤0.0001	0.238	0.128 to 0.341	≤0.0001	0.195	0.102 to 0.284	≤0.0001
IL-12p70	0.006	-0.106 to 0.119	0.9101	-0.063	-0.174 to 0.050	0.2614	0.262	0.172 to 0.348	≤0.0001
IFN-γ	0.047	-0.066 to 0.159	0.3969	-0.025	-0.137 to 0.088	0.6575	0.174	0.081 to 0.264	0.0002
IL-10	0.042	-0.071 to 0.154	0.4521	-0.040	-0.151 to 0.074	0.4856	0.253	0.163 to 0.340	≤0.0001
Active TGF-β	0.067	-0.046 to 0.178	0.2308	-0.043	-0.155 to 0.070	0.4405	0.175	0.082 to 0.265	0.0002

[a]Spearman correlation Bonferroni corrected ***p≤0.0029***

Table S3. Informative Variable Effect Sizes in LAUREL Nested Follow-up Cohort at Baseline (Prior to SLE Transition)

		Rel vs SLE			Rel vs ILE			ILE vs SLE		
**Variable**	**Variable Category**	**Odds Ratio[a]**	**95% CI**	***p-value[b]***	**Odds Ratio[a]**	**95% CI**	***p-value[b]***	**Odds Ratio[a]**	**95% CI**	***p-value[b]***
Chronic Fatigue	Type II Symptoms (Self-reported)	13.11	6.19-27.1	***2.10E-13***	3.61	1.69-7.40	***1.77E-03***	3.63	1.43-8.88	***1.41E-02***
Anixiety	Type II Symptoms (Self-reported)	2.74	1.41-5.31	***3.07E-03***	3.02	1.35-6.62	***5.68E-03***	1.10	0.46-2.61	*8.31E-01*
Depression	Type II Symptoms (Self-reported)	3.24	1.67-3.01	***2.77E-04***	3.30	1.56-7.27	***3.38E-03***	1.02	0.41-2.43	*1.00E+00*
Chronic Headaches	Type II Symptoms (Self-reported)	2.28	1.19-4.18	***1.24E-02***	1.81	0.88-3.68	*1.33E-01*	1.26	0.53-3.09	*6.57E-01*
Sleep <7h/night	Type II Symptoms (Self-reported)	3.77	1.95-7.15	***9.17E-05***	1.97	0.93-4.31	*9.54E-02*	1.91	0.78-4.41	*1.85E-01*
Malar Rash	ACR/mSLERPI[c]	24.8	3.59-277	***1.42E-04***	19.4	0.30-238	***4.64E-03***	1.28	0.39-4.07	*7.63E-01*
Discoid	ACR/mSLERPI[c]	5.54	0.63-80.6	*1.79E-01*	9.12	1.02-133	*9.08E-02*	1.65	0.25-10.8	*1.00E+00*
Photosensitivity	ACR[c]	50.5	7.69-538	***3.14E-08***	44.3	6.11-494	***5.96E-06***	1.14	0.43-3.14	*1.00E+00*
Oral Ulcers	ACR/mSLERPI[c]	8.46	1.23-110	*6.17E-02*	9.12	1.02-133	*9.08E-02*	1.08	0.18-5.50	*1.00E+00*
Arthritis	ACR/mSLERPI[c]	88.0	14.6-919	***9.75E-13***	68.2	10.78-739	***2.57E-08***	1.29	0.53-3.14	*6.57E-01*
Serositis	ACR/mSLERPI[c]	14.6	1.91-173	***6.08E-03***	4.43	0.23-84.5	*3.41E-01*	3.30	0.41-39.9	*4.01E-01*
Renal	ACR[c]	5.54	0.63-80.6	*1.79E-01*	9.12	1.02-133	*9.08E-02*	1.65	0.25-10.8	*6.36E-01*
Proteinuria	mSLERPI[c]	5.54	0.63-80.6	*1.79E-01*	9.12	1.02-133	*9.08E-02*	1.65	0.25-10.8	*6.36E-01*
Neurologic	ACR/mSLERPI[c]	5.54	0.63-80.6	*1.79E-01*	4.43	0.23-84.5	*3.41E-01*	1.25	0.14-18.6	*1.00E+00*
Hematologic	ACR[c]	15.0	1.96-178	***5.60E-03***	32.8	4.88-378	***1.44E-04***	2.19	0.60-7.09	*3.20E-01*
Leukopenia	mSLERPI[c]	8.66	1.25-113	*5.89E-02*	19.1	2.94-235	***4.83E-03***	2.21	0.56-9.13	*4.26E-01*
Thrombocytopenia or Hemolytic Anemia	mSLERPI[c]	--	--	--	--	--	--	--	--	--
Immunologic	ACR/mSLERPI[c]	3.28	1.70-6.52	***6.68E-04***	2.76	1.27-5.98	***1.28E-02***	1.19	0.47-2.79	*8.21E-01*
ANA	ACR/mSLERPI[c]	9.94	3.94-23.9	***2.21E-08***	7.31	2.61-19.9	***3.78E-05***	1.36	0.39-5.08	*7.25E-01*
SLE-CSQ (Probable)[d]	SLE-CSQ	13.6	6.39-28.1	***1.22E-13***	5.41	2.49-12.2	***2.63E-05***	2.51	0.92-6.27	*7.87E-02*
IL-12p70 (≥31.65 pg/ml)[e]	Immune Mediator (Th1-like)	2.57	1.32-5.11	***6.76E-03***	2.05	0.95-4.45	*8.69E-02*	1.25	0.50-3.19	*8.06E-01*
IFN-γ (≥86.27 pg/ml)[e]	Immune Mediator (Th1-like)	4.50	1.91-10.5	***4.62E-04***	1.30	0.59-2.84	*6.90E-01*	3.47	1.15-11.2	***4.39E-02***
IL-2Rα (≥819.5 pg/ml)[e]	Immune Mediator (Th1-like)	2.85	1.46-5.54	***1.81E-03***	1.93	0.84-4.27	*1.24E-01*	1.48	0.62-3.47	*5.08E-01*
Active TGF-β (≥819.5 pg/ml)[e]	Immune Mediator (Regulatory)	3.19	1.47-6.45	***1.81E-03***	1.45	0.68-3.13	*4.39E-01*	2.20	0.77-5.57	*1.30E-01*
IL-10 (≥39.96 pg/ml)[e]	Immune Mediator (Regulatory)	4.21	1.62-10.3	***1.74E-03***	1.01	0.45-2.24	*1.00E+00*	4.25	1.31-12.2	***1.83E-02***
MCP-1/CCL2 (≥165.6 pg/ml)[e]	Immune Mediator (IFN-associated chemokine)	2.54	1.24-5.06	***9.39E-03***	1.92	0.87-4.58	*1.25E-01*	1.32	0.50-3.69	*6.14E-01*
MCP-3/CCL7 (≥4424 pg/ml)[e]	Immune Mediator (IFN-associated chemokine)	4.38	2.19-8.43	***2.46E-05***	1.69	0.71-3.81	*2.35E-01*	2.59	1.02-6.12	***4.83E-02***
IP-10/CXCL10 (≥1153 pg/ml)[e]	Immune Mediator (IFN-associated chemokine)	1.86	0.98-3.51	*6.13E-02*	1.07	0.50-2.21	*1.00E+00*	1.74	0.73-4.26	*2.74E-01*
BLyS (≥832.1 pg/ml)[e]	Immune Mediator (TNF superfamily)	3.68	1.98-6.81	***5.78E-05***	2.48	1.18-5.38	***2.77E-02***	1.48	0.61-3.60	*3.87E-01*
TNF-α (≥35.32 pg/ml)[e]	Immune Mediator (TNF superfamily)	3.15	1.41-6.73	***5.54E-03***	1.04	0.47-2.20	*1.00E+00*	3.27	1.12-9.60	***3.47E-02***
TNFRI (≥1844 pg/ml)[e]	Immune Mediator (TNF superfamily)	2.48	1.25-4.96	***1.01E-02***	1.19	0.56-2.46	*7.05E-01*	2.94	1.12-7.33	***2.23E-02***
TNFRII (≥5063 pg/ml)[e]	Immune Mediator (TNF superfamily)	2.47	1.29-4.75	***1.17E-02***	1.17	0.50-2.88	*8.17E-01*	2.10	0.79-5.51	*1.67E-01*
SCF (≥486.1 pg/ml)[e]	Immune Mediator (Other inflammatory)	9.81	4.55-21.9	***1.84E-10***	2.49	1.19-5.32	***1.95E-02***	3.95	1.50-10.3	***6.89E-03***
anti-dsDNA[f]	SLE-associated AutoAb	3.43	1.20-11.0	***4.69E-02***	1.40	0.22-16.5	*1.00E+00*	4.80	0.78-55.5	*1.48E-01*
anti-Chromatin[f]	SLE-associated AutoAb	1.23	0.48-3.18	*6.26E-01*	1.15	0.39-3.59	*7.64E-01*	1.07	0.30-3.49	*1.00E+00*
anti-Ro/SSA[f]	SLE-associated AutoAb	3.09	1.40-6.69	***6.83E-03***	3.34	1.39-7.93	***1.89E-02***	1.08	0.42-2.82	*1.00E+00*
anti-La/SSB[f]	SLE-associated AutoAb	2.96	0.87-9.96	*8.77E-02*	4.25	1.34-15.9	***2.96E-02***	1.44	0.44-5.09	*7.41E-01*
anti-Sm[f]	SLE-associated AutoAb	8.46	1.23-10.4	*6.17E-02*	4.43	0.23-84.5	*3.41E-01*	1.91	0.27-25.4	*1.00E+00*
anti-SmRNP[f]	SLE-associated AutoAb	2.89	0.81-10.1	*2.13E-01*	1.14	0.09-7.24	*1.00E+00*	2.54	0.39-31.9	*6.46E-01*
anti-RNP[f]	SLE-associated AutoAb	3.50	1.17-9.24	***2.90E-02***	1.57	0.27-18.2	*1.00E+00*	5.50	0.75-62.7	*1.45E-01*

[a]Fisher Exact test with Haldone-Anscombe correction

[b]Bonferroni multiple comparison adjusted significant ***p≤0.00125***

[c]1997 ACR Classification and/or modified SLE Risk Probability Index (mSLERPI) criteria

[d]SLE-CSQ score ≥4

[e]Cut-off determined by Youdin Index (Rel vs. SLE)

[f]Determined by Bioplex 2200 multipex assay

Table S4. Informative Variable Effect Sizes in LAUREL Nested Follow-up Cohort at Follow-up (After SLE Transition)

		Rel vs SLE			Rel vs ILE			ILE vs SLE		
**Variable**	**Variable Category**	**Odds Ratio[a]**	**95% CI**	***p-value*[b]**	**Odds Ratio[a]**	**95% CI**	***p-value*[b]**	**Odds Ratio[a]**	**95% CI**	***p-value*[b]**
Chronic Fatigue	Type II Symptoms (Self-reported)	8.54	4.16-16.6	***3.56E-10***	4.17	1.96-8.96	***2.78E-04***	2.05	0.82-5.15	*1.54E-01*
Anixiety	Type II Symptoms (Self-reported)	2.55	1.30-4.94	***5.49E-03***	3.02	1.35-6.62	***5.68E-03***	1.19	0.49-2.82	*8.27E-01*
Depression	Type II Symptoms (Self-reported)	2.34	1.23-4.30	***8.09E-03***	1.58	0.76-3.37	*2.55E-01*	1.48	0.61-3.60	*3.87E-01*
Chronic Headaches	Type II Symptoms (Self-reported)	4.10	2.19-7.07	***1.38E-05***	2.28	1.07-4.87	***4.43E-02***	1.80	0.75-4.45	*1.94E-01*
Sleep <7h/night	Type II Symptoms (Self-reported)	1.42	0.76-2.64	*3.32E-01*	3.05	1.32-6.89	***8.42E-03***	0.46	0.19-1.21	*1.16E-01*
Malar Rash	ACR/mSLERPI[c]	220	37.0-2255	***<1.00E-15***	31.0	4.64-357	***1.83E-04***	7.08	2.51-19.5	***8.57E-05***
Discoid	ACR/mSLERPI[c]	36.3	6.07-394	***2.59E-06***	9.12	1.02-133	*9.08E-02*	3.98	0.95-18.7	*1.22E-01*
Photosensitivity	ACR[c]	164	27.6-1696	***<1.00E-15***	55.1	8.00-607	***6.89E-07***	2.98	1.18-7.07	***2.73E-02***
Oral Ulcers	ACR/mSLERPI[c]	126	21.3-1305	***<1.00E-15***	31.0	4.64-357	***1.83E-04***	4.06	1.43-11.2	***6.86E-03***
Arthritis	ACR/mSLERPI[c]	459	71.8-4680	***<1.00E-15***	194	27.7-2028	***<1.00E-15***	2.37	0.97-5.73	*6.77E-02*
Serositis	ACR/mSLERPI[c]	126	21.3-1305	***<1.00E-15***	9.12	1.02-133	*9.08E-02*	13.81	3.50-61.5	***2.56E-05***
Renal	ACR[c]	17.9	2.78-206	***1.79E-03***	9.12	1.02-133	*9.08E-02*	1.96	0.45-9.90	*7.06E-01*
Proteinuria	mSLERPI[c]	17.9	2.78-206	***1.79E-03***	9.12	1.02-133	*9.08E-02*	2.98	1.18-7.07	***2.73E-02***
Neurologic	ACR/mSLERPI[c]	10.9	2.26-52.4	***1.61E-03***	1.46	0.11-10.0	*5.67E-01*	5.60	0.76-63.8	*1.46E-01*
Hematologic	ACR[c]	25.5	3.68-285	***1.23E-04***	47.1	6.44-525	***4.28E-06***	1.85	0.58-5.43	*2.73E-01*
Leukopenia	mSLERPI[c]	15.0	1.96-178	***5.60E-03***	26.4	3.30-312	***7.81E-04***	1.76	0.51-6.05	*4.94E-01*
Thrombocytopenia or Hemolytic Anemia	mSLERPI[c]	5.54	0.63-80.6	*1.79E-01*	14.1	2.01-184	***2.14E-02***	2.545	0.49-14.8	*3.68E-01*
Immunologic	ACR/mSLERPI[c]	2.23	1.13-4.16	***1.17E-02***	2.91	1.39-6.14	***6.70E-03***	1.30	0.55-8.96	*6.61E-01*
ANA	ACR/mSLERPI[c]	15.4	3.89-66.1	***4.02E-07***	18.9	3.42-196	***2.54E-05***	1.22	0.14-18.2	*1.00E+00*
SLE-CSQ (Probable)[d]	SLE-CSQ	17.7	7.71-37.8	***2.00E-15***	8.19	3.55-19.5	***3.33E-07***	2.16	0.74-6.70	*1.73E-01*
IL-12p70 (≥131.8 pg/ml)[e]	Immune Mediator (Th1-like)	11.0	3.56-34.6	***8.06E-07***	1.14	0.54-2.50	*8.46E-01*	9.64	2.52-33.5	***3.65E-04***
IFN-γ (≥90.8 pg/ml)[e]	Immune Mediator (Th1-like)	8.07	2.19-19.5	***5.83E-07***	1.90	0.88-4.12	*1.28E-01*	4.25	1.31-12.2	***1.83E-02***
IL-2Rα (≥499.3 pg/ml)[e]	Immune Mediator (Th1-like)	3.80	2.01-7.48	***4.41E-05***	2.10	1.01-4.58	*5.63E-02*	1.81	0.72-4.17	*2.56E-01*
Active TGF-β (≥183.7 pg/ml)[e]	Immune Mediator (Regulatory)	14.0	3.71-60.8	***4.60E-06***	1.40	0.55-3.49	*4.53E-01*	19.70	3.90-95.4	***9.95E-05***
IL-10 (≥31.15 pg/ml)[e]	Immune Mediator (Regulatory)	11.0	3.56-34.6	***8.06E-07***	1.00	0.48-2.11	*1.00E+00*	10.94	2.93-37.8	***1.30E-04***
MCP-1/CCL2 (≥168.7 pg/ml)[e]	Immune Mediator (IFN-associated chemokine)	2.42	1.27-4.62	***7.35E-03***	2.24	1.08-4.56	***3.72E-02***	1.08	0.46-2.61	*1.00E+00*
MCP-3/CCL7 (≥3911 pg/ml)[e]	Immune Mediator (IFN-associated chemokine)	3.52	0.87-6.82	***1.19E-04***	1.46	0.68-3.24	*3.90E-01*	2.41	1.00-5.91	*8.00E-02*
IP-10/CXCL10 (≥1107 pg/ml)[e]	Immune Mediator (IFN-associated chemokine)	2.05	1.07-3.82	***2.90E-02***	1.33	0.65-2.89	*5.70E-01*	1.54	0.62-3.81	*3.75E-01*
BLyS (≥763.9 pg/ml)[e]	Immune Mediator (TNF superfamily)	2.68	1.40-4.89	***2.70E-03***	2.30	1.11-4.69	***3.49E-02***	1.17	0.49-2.83	*8.24E-01*
TNF-α (≥31.92 pg/ml)[e]	Immune Mediator (TNF superfamily)	4.15	1.93-8.37	***1.05E-04***	1.01	0.49-2.15	*1.00E+00*	4.09	1.63-9.99	***4.18E-03***
TNFRI (≥1521 pg/ml)[e]	Immune Mediator (TNF superfamily)	2.24	1.20-4.36	***1.82E-02***	1.57	0.76-3.29	*2.61E-01*	1.42	0.60-3.34	*4.94E-01*
TNFRII (≥3484 pg/ml)[e]	Immune Mediator (TNF superfamily)	1.91	1.05-3.52	***4.15E-02***	1.31	0.57-2.71	*5.61E-01*	1.46	0.62-3.57	*5.15E-01*
SCF (≥439.2 pg/ml)[e]	Immune Mediator (Other inflammatory)	6.43	3.05-14.2	***2.94E-07***	1.56	0.76-3.39	*2.60E-01*	4.12	1.57-10.8	***6.18E-03***
anti-dsDNA[f]	SLE-associated AutoAb	1.62	0.51-5.36	*4.89E-01*	1.57	0.27-18.2	*1.00E+00*	2.54	0.39-31.9	*6.46E-01*
anti-Chromatin[f]	SLE-associated AutoAb	7.45	1.51-37.9	***1.56E-02***	7.36	1.43-42.1	***4.20E-02***	1.01	0.24-4.04	*1.00E+00*
anti-Ro/SSA[f]	SLE-associated AutoAb	2.95	1.39-6.54	***8.36E-03***	2.48	0.97-6.50	*8.88E-02*	1.19	0.45-3.28	*8.07E-01*
anti-La/SSB[f]	SLE-associated AutoAb	2.96	0.87-9.96	*8.77E-02*	4.25	1.34-15.9	***2.96E-02***	1.44	0.44-5.09	*7.41E-01*
anti-Sm[f]	SLE-associated AutoAb	5.67	0.64-82.5	*1.74E-01*	2.20	0.15-1932	*4.66E-01*	1.91	0.27-25.4	*1.00E+00*
anti-SmRNP[f]	SLE-associated AutoAb	4.50	1.13-14.5	***2.40E-02***	1.14	0.09-7.24	*1.00E+00*	3.96	0.58-46.7	*2.47E-01*
anti-RNP[f]	SLE-associated AutoAb	2.52	0.78-7.15	*1.13E-01*	1.31	0.27-6.44	*6.67E-01*	1.92	0.44-9.80	*7.05E-01*

[a]Fisher Exact test with Haldone-Anscombe correction

[b]Bonferroni multiple comparison adjusted significant ***p≤0.00125***

[c]1997 ACR Classification and/or modified SLE Risk Probability Index (mSLERPI) criteria

[d]SLE-CSQ score ≥4

[e]Cut-off determined by Youdin Index (Rel vs. SLE)

[f]Determined by Bioplex 2200 multipex assay

Table S5. Informative Variable Effect Sizes in LFRR Nested Cohort (After SLE Transition)

		Rel vs SLE			Rel vs ILE			ILE vs SLE		
**Variable**	**Variable Category**	**Odds Ratio[a]**	**95% CI**	***p-value*[b]**	**Odds Ratio[a]**	**95% CI**	***p-value*[b]**	**Odds Ratio[a]**	**95% CI**	***p-value*[b]**
Chronic Fatigue	Type II Symptoms (Self-reported)	8.92	5.05-15.4	***2.00E-15***	10.67	5.45-20.1	***3.50E-14***	1.20	0.60-2.35	*7.24E-01*
Anixiety	Type II Symptoms (Self-reported)	1.97	1.10-3.39	***2.02E-02***	1.55	0.85-2.86	*2.04E-01*	1.55	0.85-2.86	*2.04E-01*
Depression	Type II Symptoms (Self-reported)	4.05	2.38-6.90	***2.06E-07***	3.23	1.79-5.81	***7.95E-05***	1.25	0.68-2.31	*5.24E-01*
Chronic Headaches	Type II Symptoms (Self-reported)	3.18	1.90-5.28	***1.14E-05***	2.80	1.56-4.96	***4.73E-04***	1.13	0.63-2.05	*7.54E-01*
Sleep <7h/night	Type II Symptoms (Self-reported)	1.67	1.01-2.78	*5.33E-02*	1.75	0.97-3.23	*7.00E-02*	1.05	0.56-1.99	*1.00E+00*
Malar Rash	ACR/mSLERPI[c]	87	14.5-897	***<1.00E-15***	37.3	5.83-399	***4.22E-07***	2.34	1.13-4.88	***1.86E-02***
Discoid	ACR/mSLERPI[c]	23.4	3.64-251	***2.56E-05***	16.72	2.76-189	***1.49E-03***	1.40	0.56-3.60	*6.32E-01*
Photosensitivity	ACR[c]	87	14.5-897	***<1.00E-15***	16.7	2.76-189	***1.30E-14***	1.05	0.57-1.93	*6.32E-01*
Oral Ulcers	ACR/mSLERPI[c]	55	9.9-568	***4.12E-11***	14.1	2.23-163	***4.41E-03***	3.88	1.49-9.57	***2.99E-03***
Arthritis	ACR/mSLERPI[c]	335	55.2-3394	***<1.00E-15***	97	17.2-1007	***3.00E-15***	3.44	1.81-6.52	***1.15E-04***
Serositis	ACR/mSLERPI[c]	95	15.9-975	***<1.00E-15***	19.39	2.86-217	***3.00E-15***	4.90	2.10-10.6	***6.03E-05***
Renal	ACR[c]	153.8	26.0-1570	***<1.00E-15***	6.76	0.99-88.0	*9.36E-02*	22.76	7.15-71.8	***1.10E-11***
Proteinuria	mSLERPI[c]	147.9	25.0-1510	***<1.00E-15***	6.76	0.99-88.0	*9.36E-02*	21.88	6.87-69.1	***2.81E-11***
Neurologic	ACR/mSLERPI[c]	25.5	4.06-272	***9.78E-06***	4.44	0.51-64.7	*2.34E-01*	5.73	1.44-25.9	***1.51E-02***
Hematologic	ACR[c]	187.2	31.6-1907	***<1.00E-15***	231.1	36.1-2390	***<1.00E-15***	1.23	0.60-2.47	*5.91E-01*
Leukopenia	mSLERPI[c]	69.9	11.5-721	***1.54E-13***	47.7	7.84-504	***9.84E-09***	1.46	0.72-2.81	*3.07E-01*
Thrombocytopenia or Hemolytic Anemia	mSLERPI[c]	51.95	8.29-540	***1.22E-10***	2.3	0.12-43.0	***9.84E-09***	23.05	4.04-241	***6.47E-06***
Immunologic	ACR/mSLERPI[c]	26.55	11.3-60.0	***<1.00E-15***	1.52	0.87-2.64	*1.51E-01*	17.51	6.81-42.9	***2.64E-12***
ANA	ACR/mSLERPI[c]	13.2	6.33-27.8	***1.00E-15***	17.5	6.67-42.0	***5.70E-14***	1.33	0.43-3.66	*7.80E-01*
SLE-CSQ (Probable)[d]	SLE-CSQ	68.0	29.0-148	***<1.00E-15***	29.57	13.3-64.4	***<1.00E-15***	2.30	0.91-5.88	*9.45E-02*
IL-12p70 (≥0.695 pg/ml)[e]	Immune Mediator (Th1-like)	3.5	2.00-6.20	***9.99E-06***	2.69	1.46-4.97	***2.61E-03***	1.31	0.69-2.42	*4.35E-01*
IFN-γ (≥1.405 pg/ml)[e]	Immune Mediator (Th1-like)	1.47	0.89-2.40	*1.56E-01*	4.59	2.41-8.49	***1.69E-06***	6.76	3.34-13.7	***1.62E-08***
IL-2Rα (≥14765 pg/ml)[e]	Immune Mediator (Th1-like)	4.09	2.40-6.96	***1.50E-07***	1.87	1.04-3.32	***4.73E-02***	2.19	1.16-4.04	***1.37E-02***
Active TGF-β (≥136.5 pg/ml)[e]	Immune Mediator (Regulatory)	2.6	0.42-31.8	*6.52E-01*	2.24	0.64-7.86	*2.65E-01*	5.77	0.92-71.2	*1.63E-01*
IL-10 (≥0.220 pg/ml)[e]	Immune Mediator (Regulatory)	4.0	2.34-6.80	***2.55E-07***	2.32	1.30-4.09	***4.27E-03***	1.73	0.91-3.30	*1.03E-01*
MCP-1/CCL2 (≥83.71 pg/ml)[e]	Immune Mediator (IFN-associated chemokine)	1.30	0.77-2.16	*3.59E-01*	2.12	1.19-2.67	***9.85E-03***	1.63	0.87-2.95	*1.24E-01*
MCP-3/CCL7 (≥175.5 pg/ml)[e]	Immune Mediator (IFN-associated chemokine)	2.82	1.61-4.86	***2.35E-04***	9.89	4.41-23.7	***2.84E-10***	27.86	11.5-67.9	***<1.00E-15***
IP-10/CXCL10 (≥119.7 pg/ml)[e]	Immune Mediator (IFN-associated chemokine)	3.97	2.31-6.76	***6.72E-07***	1.53	0.82-2.88	***4.73E-02***	2.60	1.39-4.92	***4.52E-03***
BLyS (≥830.7 pg/ml)[e]	Immune Mediator (TNF superfamily)	13.18	6.76-25.9	***<1.00E-15***	5.74	2.66-11.8	***3.36E-06***	2.30	1.20-4.40	***1.25E-02***
TNF-α (≥15.7 pg/ml)[e]	Immune Mediator (TNF superfamily)	1.59	0.98-2.64	*7.48E-02*	3.83	1.97-7.40	***5.69E-05***	6.11	2.88-12.6	***2.82E-07***
TNFRI (≥5171 pg/ml)[e]	Immune Mediator (TNF superfamily)	4.00	2.28-6.98	***4.62E-07***	2.00	1.11-3.75	***3.55E-02***	2.00	1.06-3.72	***3.10E-02***
TNFRII (≥950.2 pg/ml)[e]	Immune Mediator (TNF superfamily)	3.00	1.74-5.08	***9.17E-05***	1.22	0.63-2.34	*6.09E-01*	2.46	1.28-4.85	***1.00E-02***
SCF (≥45.14 pg/ml)[e]	Immune Mediator (Other inflammatory)	2.01	1.03-4.20	*5.22E-02*	22.37	3.76-231	***2.61E-06***	11.61	1.81-125	***4.57E-03***
anti-dsDNA[f]	SLE-associated AutoAb	23.9	9.35-57.6	***<1.00E-15***	2.32	0.31-27.7	*6.68E-01*	55.51	9.03-572	***1.17E-11***
anti-Chromatin[f]	SLE-associated AutoAb	14.2	6.64-28.5	***3.00E-15***	3.90	1.68-9.10	***2.25E-03***	3.65	1.82-7.28	***2.01E-04***
anti-Ro/SSA[f]	SLE-associated AutoAb	6.24	3.18-12.4	***3.75E-08***	3.69	1.76-7.53	***9.09E-04***	1.69	0.87-3.32	*1.39E-01*
anti-La/SSB[f]	SLE-associated AutoAb	10.9	2.64-49.4	***2.64E-04***	14.07	3.23-64.4	***6.98E-05***	1.29	0.55-2.95	*6.52E-01*
anti-Sm[f]	SLE-associated AutoAb	41.4	11.2-478	***3.20E-14***	4.59	1.04-24.4	***6.98E-05***	9.03	3.22-24.6	***4.01E-06***
anti-SmRNP[f]	SLE-associated AutoAb	28.9	10.1-77.0	***<1.00E-15***	4.59	1.04-24.4	***6.75E-04***	4.16	1.99-9.20	***1.12E-04***
anti-RNP[f]	SLE-associated AutoAb	21.1	8.21-50.9	***1.20E-14***	5.52	1.77-14.7	***1.61E-03***	3.82	1.83-8.50	***3.45E-04***

[a]Fisher Exact test with Haldone-Anscombe correction

[b]Bonferroni multiple comparison adjusted significant ***p≤0.00125***

[c]1997 ACR Classification and/or modified SLE Risk Probability Index (mSLERPI) criteria

[d]SLE-CSQ score ≥4

[e]Cut-off determined by Youdin Index (Rel vs. SLE)

[f]Determined by Bioplex 2200 multipex assay

	**LAUREL Nested Study (BL)**<br>(Prior to SLE Transition)<br>All lupus relatives meet <4 Classification Criteria<br>(-->ILE, -->SLE + matched Rel and HC)	**LAUREL Nested Study (FU)**<br>(After SLE Transition)<br>Lupus relatives meet 0-2, 3, or ≥4 Classification Criteria<br>(ILE, SLE + matched Rel and HC)	**LFRR Confirmatory Nested Study**<br>(After SLE Transition)<br>Lupus relatives meet 0-2, 3, or ≥4 Classification Criteria<br>(ILE, SLE + matched Rel and HC)
**Entered Cohort**	December 1992 - January 2011	October 2009 - May 2012	August 1992 - August 2008
**Medical Record Review**<br>**Clinical Serology**<br>**SLE-CSQ Calculations**	January 1996 - April 2011	October 2009 - May 2012	August 1992 - September 2011
**SLE-Associated Autoantibody Specificity Determination (Bioplex 2200)**	August 2010 - February 2011	August 2010 - October 2012	September 2005 - May 2012
**SLE Classification Verification**<br>**Sample Selection/Matching**<br>**Plasma Soluble Mediator Assessment (xMAP, ELISA)**	April 2011 - October 2012	April 2011 - October 2012	May 2014 - October 2014

Figure S1. Flow chart of nested study enrollment and assessments. Dates of enrollment into parent cohorts, medical record review/clinical serology (for 1997 SLE classification criteria determination to differentiate SLE, ILE [3 criteria], and lupus relatives [Rel; 0-2 criteria]), SLE-CSQ score calculations (from questionnaire), as well as determination of SLE-associated autoantibody specificities, and selection/immune mediator assessment of matched plasma samples (Rel and HC to ILE and SLE). LAUREL nested cohort at baseline (pre-classification): →ILE (n=34), →SLE (n=56), matched (race, sex, age ± 5 years) Rel (n=154) and HC (n=77). LAUREL nested cohort at follow-up (post-classification): ILE (n=34), SLE (n=56), matched (race, sex, age ± 5 years) Rel (n=154) and HC (n=77). LFRR confirmatory nested cohort (post-classificaiton): ILE (n=72), SLE (n=100), marched (race, sex) Rel (n=159 and HC (n=127).

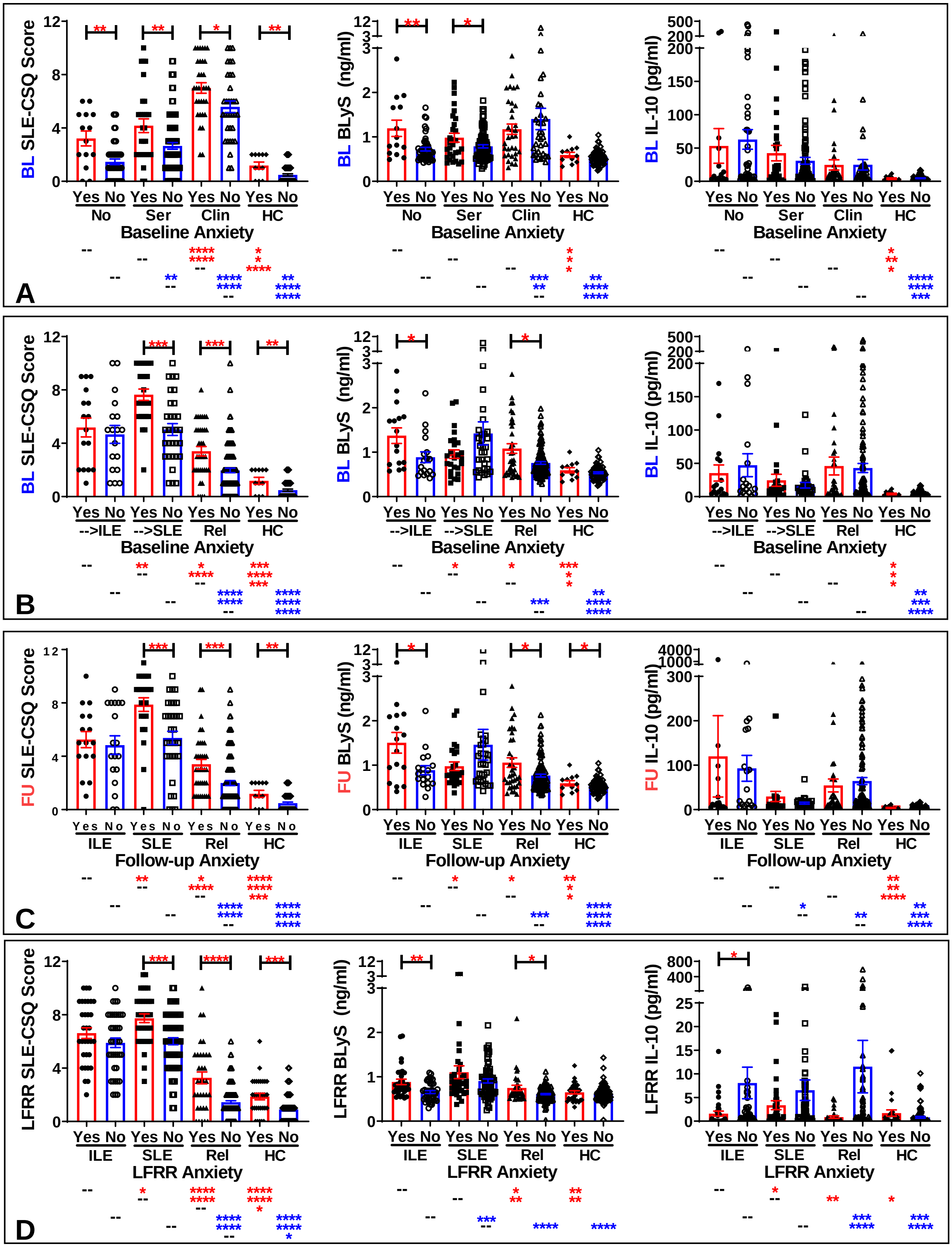
A
BL SLE-CSQ Score
BL BLyS (ng/ml)
BL IL-10 (pg/ml)
Yes No Yes No Yes No Yes No
No Ser Clin HC
Baseline Anxiety
B
BL SLE-CSQ Score
BL BLyS (ng/ml)
BL IL-10 (pg/ml)
Yes No Yes No Yes No Yes No
-->ILE -->SLE Rel HC
Baseline Anxiety
C
FU SLE-CSQ Score
FU BLyS (ng/ml)
FU IL-10 (pg/ml)
Yes No Yes No Yes No Yes No
ILE SLE Rel HC
Follow-up Anxiety
D
LFRR SLE-CSQ Score
LFRR BLyS (ng/ml)
LFRR IL-10 (pg/ml)
Yes No Yes No Yes No Yes No
ILE SLE Rel HC
LFRR Anxiety

Figure S2. Altered SLE-CSQ scores and BLyS and IL-10 levels associated with reported anxiety in lupus relatives prior to disease transition in the LAUREL cohort. Lupus relatives and matched healthy controls (HC) were evaluated for SLE-CSQ scores (*1st column*), plasma BLyS levels (*2nd column*), and plasma IL-10 levels (*4th column)* in lupus relatives vs. matched healthy controls (HC) who did (Yes) or did not (No) report anxiety on the LFRR questionnaire in (**A**) LAUREL cohort at baseline meeting No ACR criteria (No), only serologic ACR criteria (Ser), or clinical ACR criteria (Clin) vs. matched, unaffected HC and (**B-D**) lupus relatives who developed ILE (ILE), transitioned to SLE (SLE), or remained clinically unaffected (Rel) vs. matched healthy controls (HC) in (**B**) LAUREL cohort at baseline (pre-transition), (**C**) LAUREL cohort at follow-up (post-transition), and (**D**) LFRR cohort (post-transition). Mean ± SEM. ****$p<0.0001$; ***$p<0.001$; **$p<0.01$; *$p<0.05$ by Kruskal-Wallis with Dunn's multiple comparison.

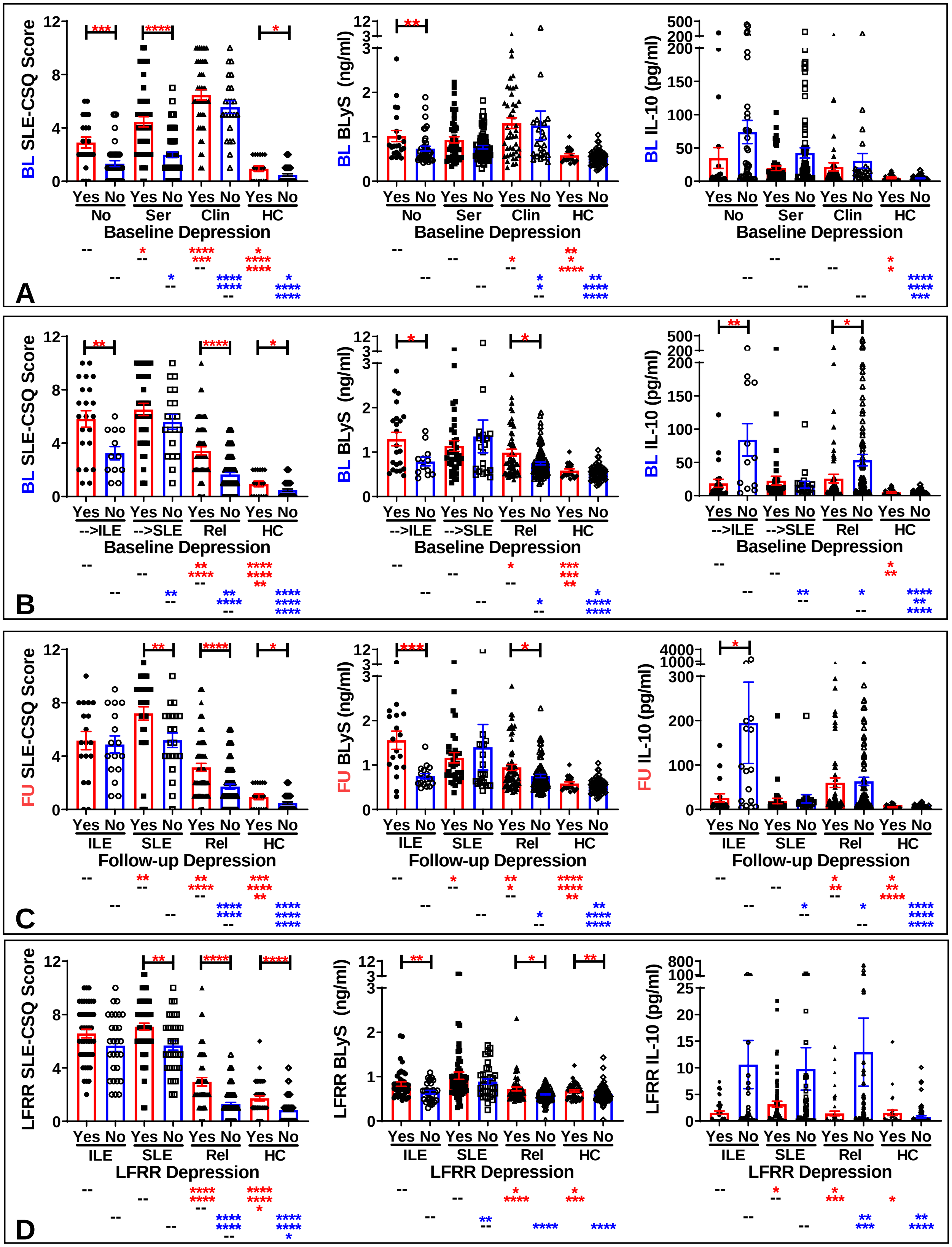
A
BL SLE-CSQ Score
BL BLyS (ng/ml)
BL IL-10 (pg/ml)
Yes No Yes No Yes No Yes No
No Ser Clin HC
Baseline Depression
B
BL SLE-CSQ Score
BL BLyS (ng/ml)
BL IL-10 (pg/ml)
Yes No Yes No Yes No Yes No
-->ILE -->SLE Rel HC
Baseline Depression
C
FU SLE-CSQ Score
FU BLyS (ng/ml)
FU IL-10 (pg/ml)
Yes No Yes No Yes No Yes No
ILE SLE Rel HC
Follow-up Depression
D
LFRR SLE-CSQ Score
LFRR BLyS (ng/ml)
LFRR IL-10 (pg/ml)
Yes No Yes No Yes No Yes No
ILE SLE Rel HC
LFRR Depression

Figure S3. Altered SLE-CSQ scores and BLyS and IL-10 levels associated with reported depression in lupus relatives prior to disease transition in the LAUREL cohort. Lupus relatives and matched healthy controls (HC) were evaluated for SLE-CSQ scores (*1st column*), plasma BLyS levels (*2nd column*), and plasma IL-10 levels (*4th column)* in lupus relatives vs. matched healthy controls (HC) who did (Yes) or did not (No) report depression on the LFRR questionnaire in (A) LAUREL cohort at baseline meeting No ACR criteria (No), only serologic ACR criteria (Ser), or clinical ACR criteria (Clin) vs. matched, unaffected HC and (B-D) lupus relatives who developed ILE (ILE), transitioned to SLE (SLE), or remained clinically unaffected (Rel) vs. matched healthy controls (HC) in (B) LAUREL cohort at baseline (pre-transition), (C) LAUREL cohort at follow-up (post-transition), and (D) LFRR cohort (post-transition). Mean ± SEM. ****$p<0.0001$; ***$p<0.001$; **$p<0.01$; *$p<0.05$ by Kruskal-Wallis with Dunn's multiple comparison.

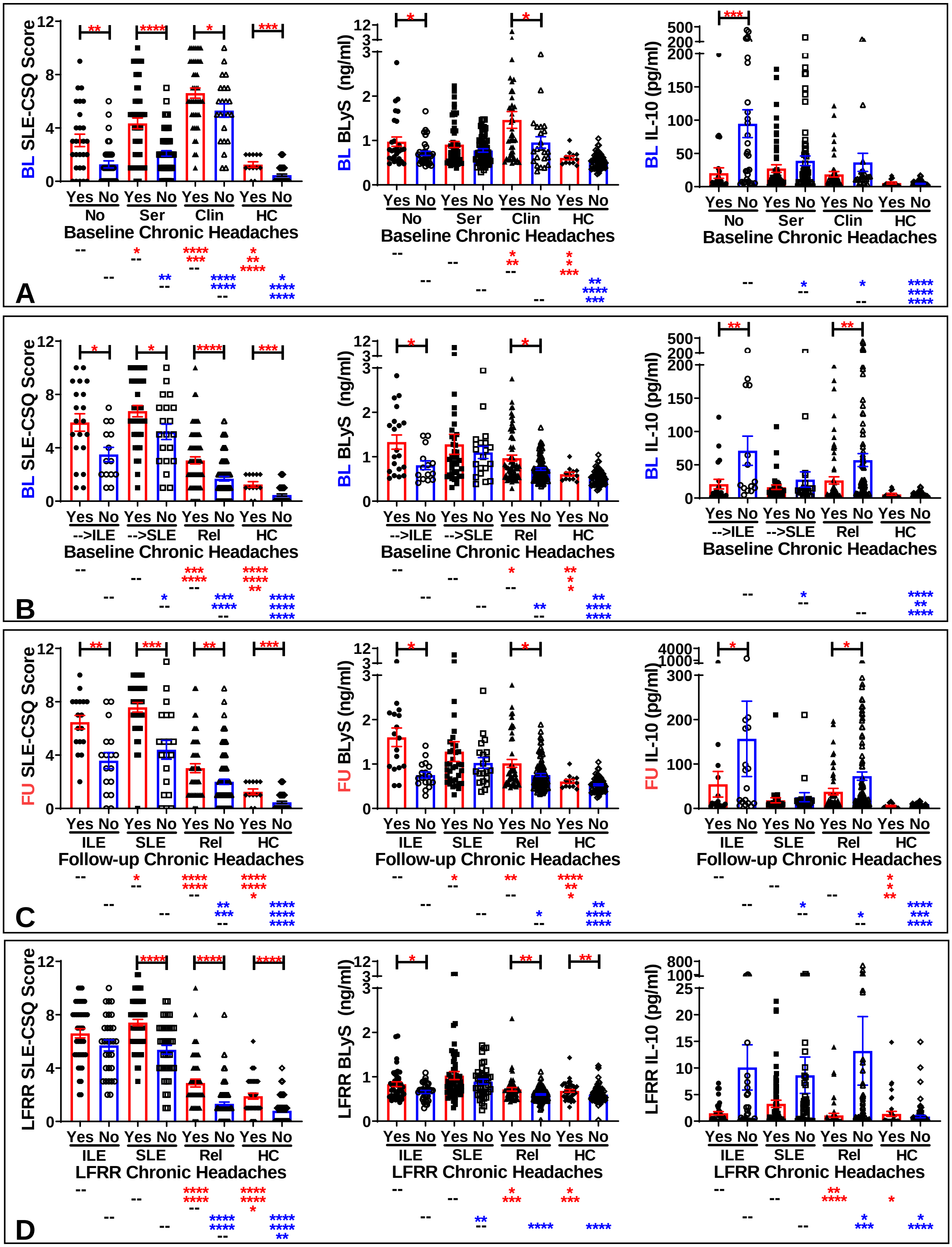
A
BL SLE-CSQ Score
Yes No Yes No Yes No Yes No
No Ser Clin HC
Baseline Chronic Headaches
BL BLyS (ng/ml)
BL IL-10 (pg/ml)
B
-->ILE -->SLE Rel HC
C
FU SLE-CSQ Score
FU BLyS (ng/ml)
FU IL-10 (pg/ml)
ILE SLE Rel HC
Follow-up Chronic Headaches
D
LFRR SLE-CSQ Score
LFRR BLyS (ng/ml)
LFRR IL-10 (pg/ml)
LFRR Chronic Headaches

Figure S4. Altered SLE-CSQ scores and BLyS and IL-10 levels associated with reported chronic headaches in lupus relatives prior to disease transition in the LAUREL cohort. Lupus relatives and matched healthy controls (HC) were evaluated for SLE-CSQ scores (*1st column*), plasma BLyS levels (*2nd column*), and plasma IL-10 levels (*4th column)* in lupus relatives vs. matched healthy controls (HC) who did (Yes) or did not (No) report chronic headaches on the LFRR questionnaire in (**A**) LAUREL cohort at baseline meeting No ACR criteria (No), only serologic ACR criteria (Ser), or clinical ACR criteria (Clin) vs. matched, unaffected HC and (**B-D**) lupus relatives who developed ILE (ILE), transitioned to SLE (SLE), or remained clinically unaffected (Rel) vs. matched healthy controls (HC) in (**B**) LAUREL cohort at baseline (pre-transition), (**C**) LAUREL cohort at follow-up (post-transition), and (**D**) LFRR cohort (post-transition). Mean $\pm$ SEM. ****$p<0.0001$; ***$p<0.001$; **$p<0.01$; *$p<0.05$ by Kruskal-Wallis with Dunn's multiple comparison.

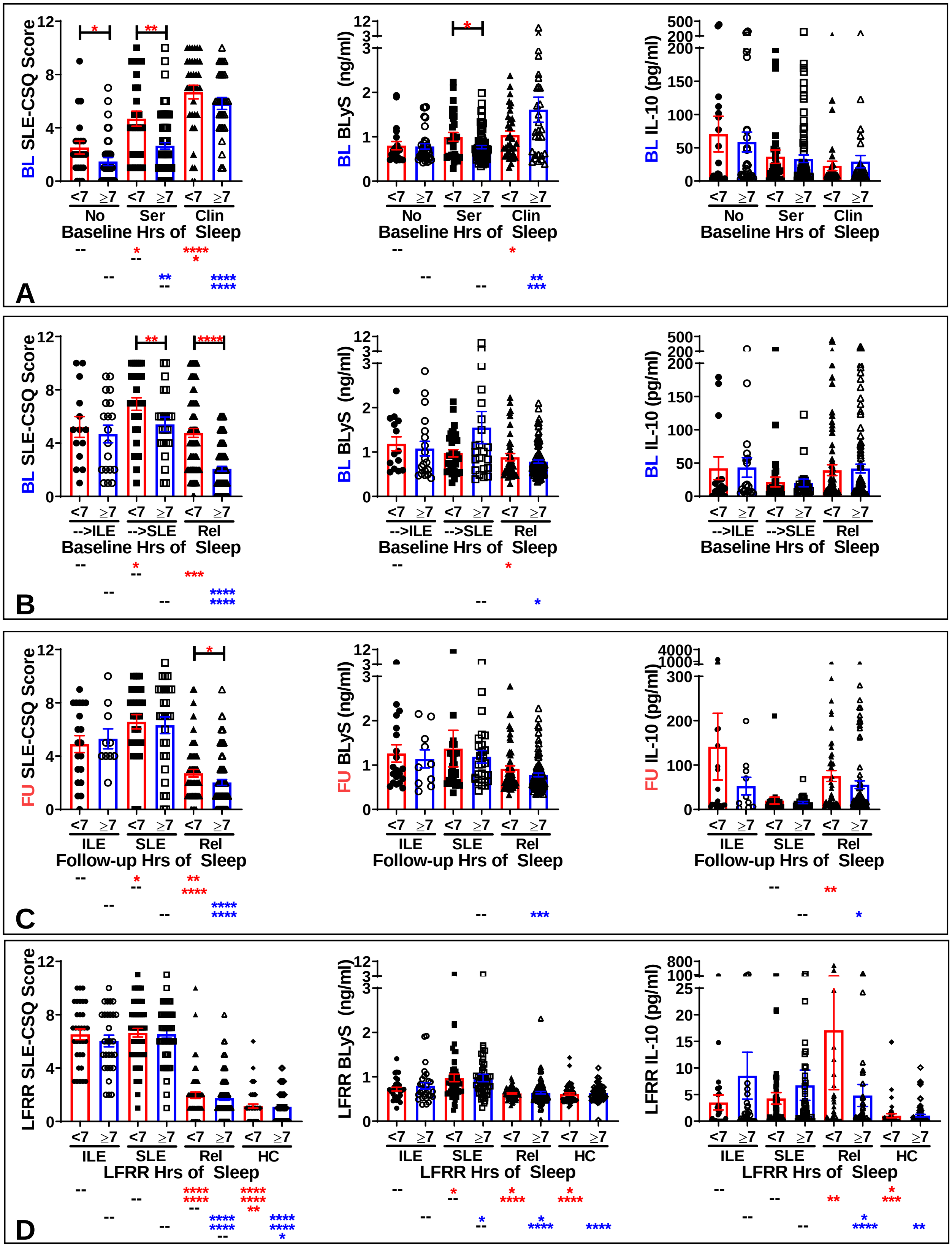
A
B
C
D
BL SLE-CSQ Score
BL BLyS (ng/ml)
BL IL-10 (pg/ml)
FU SLE-CSQ Score
FU BLyS (ng/ml)
FU IL-10 (pg/ml)
LFRR SLE-CSQ Score
LFRR BLyS (ng/ml)
LFRR IL-10 (pg/ml)
No
Ser
Clin
Baseline Hrs of Sleep
-->ILE
-->SLE
Rel
ILE
SLE
HC
Follow-up Hrs of Sleep
LFRR Hrs of Sleep
<7
≥7

Figure S5. Altered SLE-CSQ scores and BLyS and IL-10 levels associated with reported sleep distrubance in lupus relatives prior to disease transition in the LAUREL cohort. Lupus relatives and matched healthy controls (HC) were evaluated for SLE-CSQ scores (*1st column*), plasma BLyS levels (*2nd column*), and plasma IL-10 levels (*4th column)* in lupus relatives vs. matched healthy controls (HC) who reported <7 or ≥7 hours of sleep/night on the LFRR questionnaire in (**A**) LAUREL cohort at baseline meeting No ACR criteria (No), only serologic ACR criteria (Ser), or clinical ACR criteria (Clin) vs. matched, unaffected HC and (**B-D**) lupus relatives who developed ILE (ILE), transitioned to SLE (SLE), or remained clinically unaffected (Rel) vs. matched healthy controls (HC) in (**B**) LAUREL cohort at baseline (pre-transition), (**C**) LAUREL cohort at follow-up (post-transition), and (**D**) LFRR cohort (post-transition). Mean ± SEM. ****$p<0.0001$; ***$p<0.001$; **$p<0.01$; *$p<0.05$ by Kruskal-Wallis with Dunn's multiple comparison.

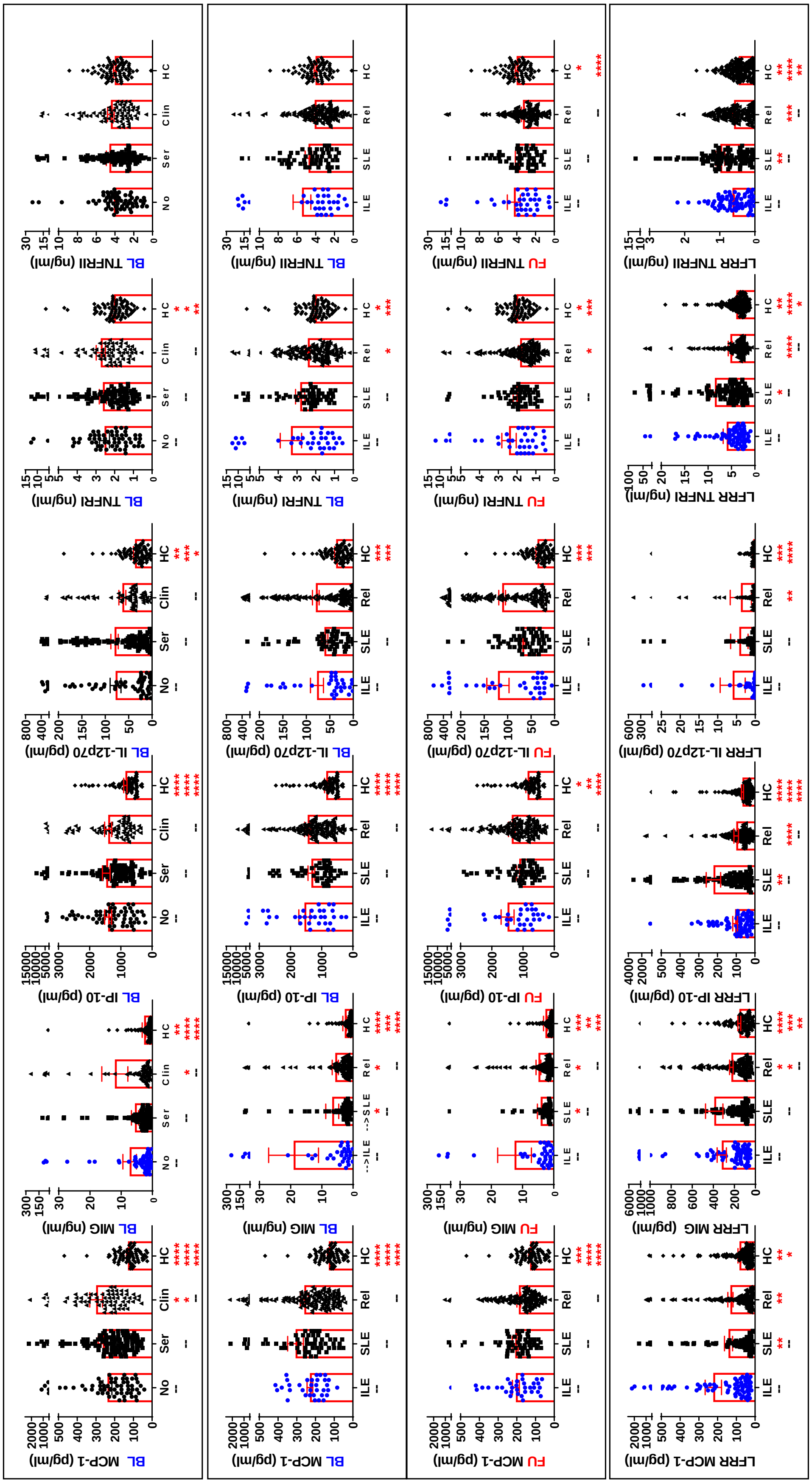

BL MCP-1 (pg/ml)
BL MIG (ng/ml)
BL IP-10 (pg/ml)
BL IL-12p70 (pg/ml)
BL TNFRI (ng/ml)
BL TNFRII (ng/ml)
No
Ser
Clin
HC
BL MCP-1 (pg/ml)
BL MIG (ng/ml)
BL IP-10 (pg/ml)
BL IL-12p70 (pg/ml)
BL TNFRI (ng/ml)
BL TNFRII (ng/ml)
ILE
SLE
Rel
HC
FU MCP-1 (pg/ml)
FU MIG (ng/ml)
FU IP-10 (pg/ml)
FU IL-12p70 (pg/ml)
FU TNFRI (ng/ml)
FU TNFRII (ng/ml)
LFRR MCP-1 (pg/ml)
LFRR MIG (pg/ml)
LFRR IP-10 (pg/ml)
LFRR IL-12p70 (pg/ml)
LFRR TNFRI (ng/ml)
LFRR TNFRII (ng/ml)

Figure S6. Altered IFN- and TNF-associated mediators in lupus relatives develop ILE or transition to SLE. Lupus relatives and matched healthy controls (HC) were evaluated for plasma levels of stem cell factor MCP-1 (*1st column*), MIG (*2nd column*), IP-10 (*3rd column*), IL-12p70 (*4th column*), soluble TNFRI (*5th column*), and soluble TNFRII (*6th column*) in (**A**) LAUREL cohort at baseline meeting No ACR criteria (No), only serologic ACR criteria (Ser), or clinical ACR criteria (Clin) vs. matched HC and (**B-D**) lupus relatives who developed ILE (ILE), transitioned to SLE (SLE), or remained clinically unaffected (Rel) vs. matched, unaffected healthy controls (HC) in (**B**) LAUREL cohort at baseline (pre-transition), (**C**) LAUREL cohort at follow-up (post-transition), and (**D**) LFRR cohort (post-transition). Mean $\pm$ SEM. ****$p<0.0001$; ***$p<0.001$; **$p<0.01$; *$p<0.05$ by Kruskal-Wallis with Dunn's multiple comparison.
